# Promising new drugs and therapeutic approaches for treatment of ovarian cancer—targeting the hallmarks of cancer

**DOI:** 10.1186/s12916-024-03826-w

**Published:** 2025-01-06

**Authors:** Julia Hillmann, Nicolai Maass, Dirk O. Bauerschlag, Inken Flörkemeier

**Affiliations:** 1 Department of Gynaecology and Obstetrics, University and University Medical Center Schleswig-Holstein Campus Kiel, Kiel, Germany; 2 Department of Gynaecology, Jena University Hospital, Jena, Germany

**Keywords:** Ovarian cancer, Therapy, Hallmarks of cancer

## Abstract

Ovarian cancer remains the most lethal gynecological malignancy. Despite the approval of promising targeted therapy such as bevacizumab and PARP inhibitors, 5-year survival has not improved significantly. Thus, there is an urgent need for new therapeutics. New advancements in therapeutic strategies target the pivotal hallmarks of cancer. This review is giving an updated overview of innovative and upcoming therapies for the treatment of ovarian cancer that focuses specific on the hallmarks of cancer. The hallmarks of cancer constitute a broad concept to reenact complexity of malignancies and furthermore identify possible targets for new treatment strategies. For this purpose, we analyzed approvals and current clinical phase III studies (registered at ClinicalTrials.gov (National Library of Medicine, National Institutes of Health; U.S. Department of Health and Human Services, 2024)) for new drugs on the basis of their mechanisms of action and identified new target approaches. A broad spectrum of new promising drugs is currently under investigation in clinical phase III studies targeting mainly the hallmarks “self-sufficiency in growth signals,” “genomic instability,” and “angiogenesis.” The benefit of immune checkpoint inhibitors in ovarian cancer has been demonstrated for the first time. Besides, targeting the tumor microenvironment is of growing interest. Replicative immortality, energy metabolism, tumor promoting inflammation, and the microbiome of ovarian cancer are still barely targeted by drugs. Nevertheless, precision medicine, which focuses on specific disease characteristics, is becoming increasingly important in cancer treatment.

**Graphical Abstract:**

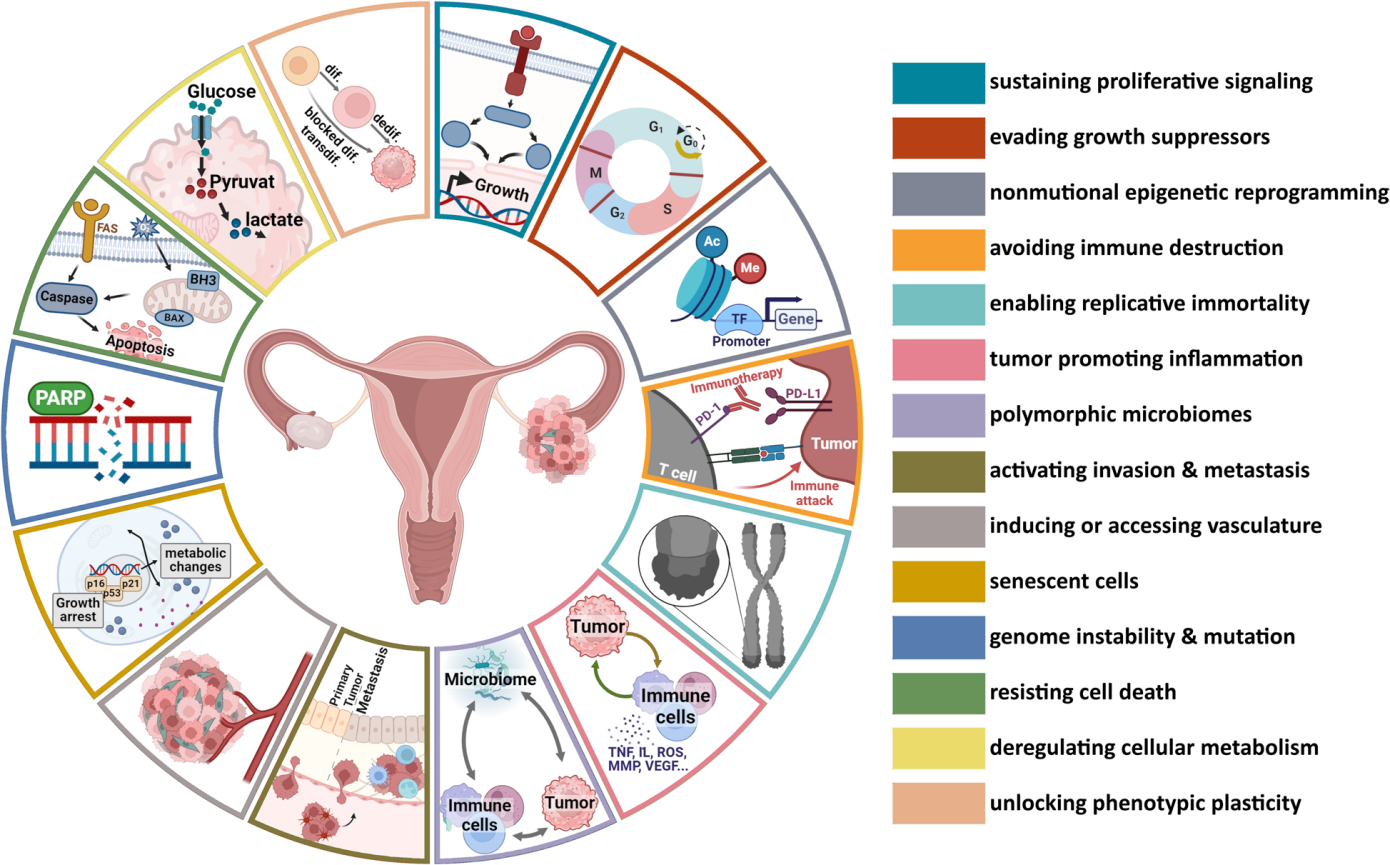

## Background

Ovarian cancer (OC) represents the 8th most common cancer death worldwide among women. In 2022, 325,000 women were newly diagnosed, and 207,000 women died from OC. With regard to the prediction of a 40.4% increase in incidence by 2045, we are facing a global problem [1–3]. Limited treatment options and late diagnosis of OC pose the major challenges. Besides the lack of biomarkers, the relapse rate is high due to natural and acquired resistance mechanisms of the cancer cells and the tumor microenvironment (TME) [4–6]. Despite innovations in treatment such as bevacizumab, poly ADP-ribose polymerase inhibitors (PARPi), and antibody drug conjugates (ADC) such as mirvetuximab soravtansine (MIRV), further therapeutic approaches are needed to significantly improve the situation.

First-line therapy for OC includes surgery followed by a chemotherapy combining platinum- and taxane-based treatment [4]. For a long time, this scheme did not undergo major changes, until bevacizumab and PARPi were supplemented for maintenance therapy. According to the SOLO1 study, olaparib was introduced as maintenance treatment of BRCA mutated (BRCAm) OC. Based on the results of PAOLA-1 study, approval of olaparib maintenance therapy was extended to combination with bevacizumab for BRCAm and BRCA-wild type (BRCAwt) but homologous recombination deficient (HRD) women [7, 8]. Niraparib is further approved as first line maintenance therapy for BRCA-wild type (BRCAwt) and homologous recombination proficient (HRP) patients. Treatment of recurrent OC (ROC) depends on platinum-responsiveness and includes combinational and single therapies with gemcitabine, liposomal doxorubicin, or topotecan. In ROC, PARPi are approved regardless of mutational status [9, 10]. Interestingly a newly developed ADC, MIRV, is approved on folate receptor alpha (FRα) overexpressing platinum resistant OC [11].

OC shows a vast tumor heterogeneity. Based on histopathological and molecular patterns, five different types of epithelial OC can be differentiated: high-grade serous cancer (HGSC), low-grade serous cancer (LGSC), mucinous cancer, endometrioid cancer, and clear cell carcinoma. So far, these differences are concomitant with slight changes in therapy algorithm. However, in order to achieve a successful response to therapy, subgroup categorization is of crucial importance. New clinical trials are increasingly focusing on the heterogenic characteristics of the tumor and using subgroups or specific biomarkers as selection criteria for inclusion in a trial, as the patient stratification influences the response to therapy and thus the success of a trial. Currently, homologous recombination deficiency (HRD) testing and next generation sequencing (NGS) is used. Four major gene mutations have been identified that are highly correlated with OC, including TP53, BRCA1/2, KRAS, and PIK3CA, resulting in abnormal DNA repair, impaired tumor suppression, gain of oncogene function, and epigenetic changes [12]. These specific characteristics can form the basis for the development of new, targeted therapies and can be classified by the hallmarks of cancer.

The introduction of the “Hallmarks of Cancer” in 2000 by Hanahan and Weinberg provides a logical framework for a better understanding of the complexity of malignant diseases and enables more systematic cancer research. The initial six hallmarks were “sustaining proliferative signaling,” “evading growth suppressors,” “resisting cell death,” “enabling replicative immortality,” “inducing angiogenesis,” and “activating invasion and metastasis,” supported by the enabling characteristics “genome instability and mutation,” and “tumor-promoting inflammation” [13]. In 2011, the hallmarks were expanded by “reprogramming energy metabolism” and “evading immune destruction.” Besides, the crucial role of TME regarding tumorigenesis and treatment response was underlined [14]. The latest update was published in January 2022 introducing two emerging hallmarks, “unlocking phenotypic plasticity,” and “senescent cells,” as well as two further enabling characteristics “nonmutational epigenetic reprogramming” and “polymorphic microbiomes” [15]. Since the hallmarks are supposed to be essential for development of cancer, described alterations in signal pathways and protein expression represent excellent targets to impair tumor growth and cancer progression, thereby not only making cancer research more logical, but also systematic drug development. This could lead to a modular system in drug development.

While classic cytostatic drugs in general inhibit increased cell proliferation and thus cause increased side effects, targeted therapy more specifically inhibits specific altered pathways of cancer cells and the microenvironment. Drug development currently focuses primarily on single receptors, while each hallmark is regulated by semi-redundant signaling pathways that allow tumor adaptation and chemoresistance through mutation. However, challenging regarding OC is the absence of a druggable driver oncogene [16, 17]. Nevertheless, many promising molecules such as kinase inhibitors, PARPi, proteasome inhibitors, or immune checkpoint inhibitors which affect altered pathways are currently investigated for OC treatment. Several other auspicious techniques, targeting among others the highly immunosuppressive TME of OC, include adoptive cell therapy, chimeric-antigen receptor T cells, cancer vaccines, and gene therapy [18, 19]. In order to prevent tumor adaptation, therapies that broadly target the hallmarks of cancer are beneficial [20].

The hallmarks of cancer describe the complexity of tumor diseases and identify potential targets for new treatment strategies. On the occasion of the last update of the hallmarks in 2022 [15] and following the review by Petrillo et al. [20] and the book chapter by El Bairi et al. [21], we would like to provide an update on the latest developments and upcoming therapeutics for the treatment of OC according to the hallmarks. This review compiles therapeutic strategies for OC based on the hallmarks of cancer and the cellular signaling pathways involved. Furthermore, promising new drugs and mechanisms of action that are investigated in actual ongoing and recently completed phase III trials are presented.

## Targeting hallmarks of cancer

### Sustaining proliferative signaling

**Fig. 1:**
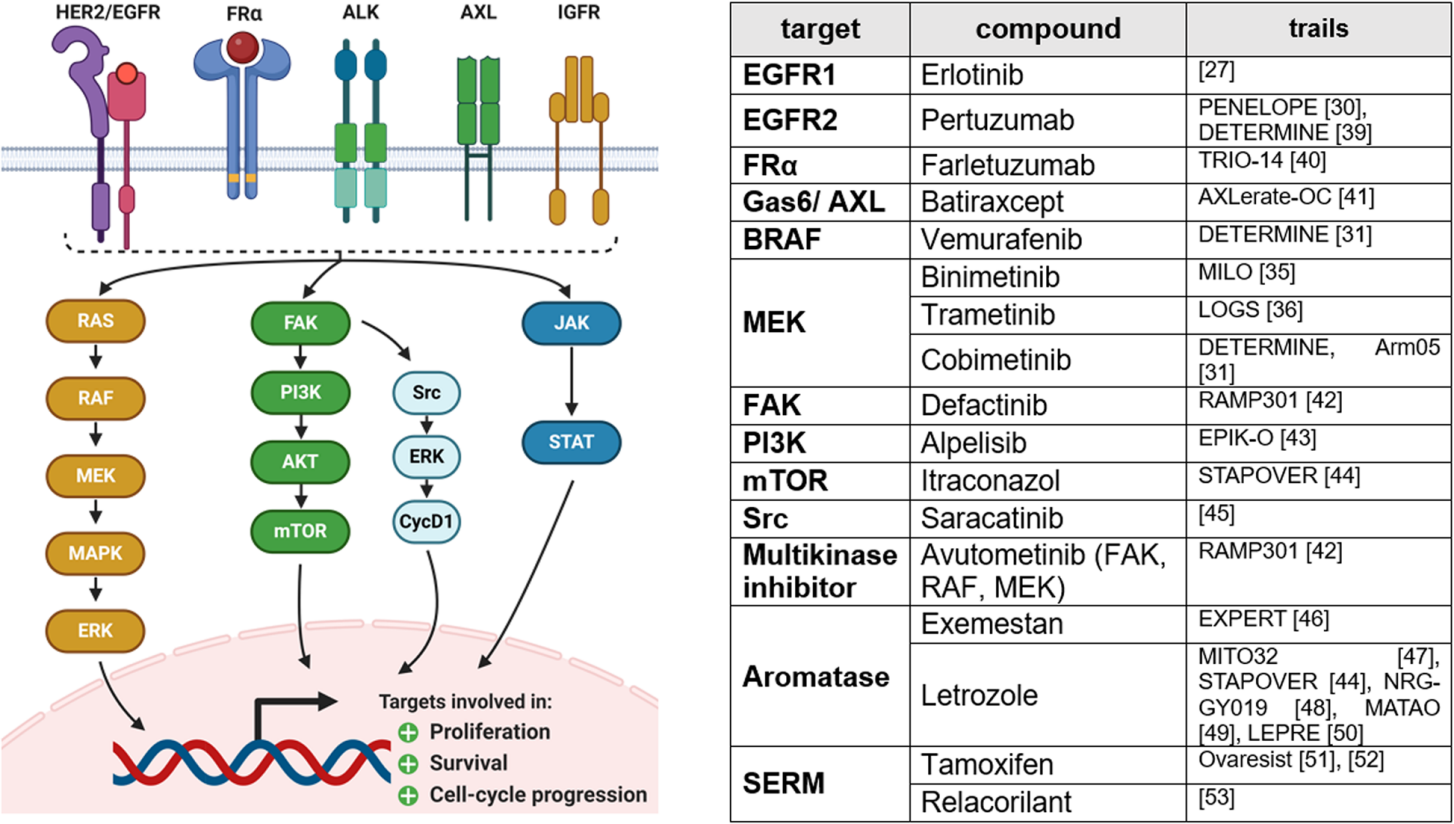
Sustaining proliferative signaling. Alterations in growth factors such as EGFR, FRα, ALK, AXL, and IGFR lead to constitutive activity in downstream signaling pathways. Most frequently affected are RAS/RAF/MAPK, PI3K/AKT/mTOR, and JAK/STAT pathway. Constitutive activity leads to dysregulated cell proliferation, cell survival, and cell cycle progression. Currently ongoing and recently completed phase III trials targeting those molecules are displayed in the table. This figure was created using Biorender.com [26, 29, 30, 34, 35, 38–52]

Deregulated cell proliferation plays a pivotal role in cancer development. Alterations in growth factors and their receptor expressions, intracellular signaling pathways, and disrupted negative-feedback mechanisms lead to constitutively activated cell proliferation [14]. Interestingly, it has been shown that extensive cell proliferation, reflected in a high expression of the oncoprotein RAS, can cause cell senescence [22]. This might play an important role regarding chemotherapy-resistance, promotion of tumor heterogeneity, and adaptive strategies becoming a more aggressive cancer [23]. Since many of the involved signal molecules are protein kinases, especially small-molecule kinase inhibitors are intensively investigated targeting this hallmark (Fig. 1).

Epidermal growth factor receptors (EGFR), such as EGFR/HER1, ErbB2/HER2, ErbB3, and ErbB4, activate multiple signaling pathways including RAS/RAF/MAPK, PI3K/AKT/mTOR, and JAK/STAT. Increased expression of EGFR was determined in 48% of OC [24, 25]. No improvement in progression-free survival (PFS) or overall survival (OS) has been demonstrated in phase III clinical trials with *erlotinib* (EGFR inhibitor) as maintenance therapy in OC [26]. Utility of EGFR localization and expression patterns as prognostic biomarker, determined by immunohistochemistry (IHC), has also been denied [27]. *Trastuzumab* and *pertuzumab*, monoclonal antibodies directed against HER2, are already successfully approved for breast cancer. HER2 expression is increased among 40% of OC [28]. Unfortunately, combination of pertuzumab and chemotherapy for OC in a phase III trial did not show benefits in PFS or OS [29]. Nevertheless, the ongoing trial DETERMINE examines the combinational treatment with trastuzumab and pertuzumab in patients with HER2 amplification and rare cancer subtype [30]. ErbB3, initially overexpressed in 41.3–67.5% of OC, concomitant with inferior prognosis and increased expression in case of recurrence, is not targeted in advanced clinical trials so far [31]. The same does apply for ErbB4, which expression is suspected to be negatively correlated with OS [32].

The RAS/RAF/MAPK pathway regulates the expression of the transcription factors, crucial for cell proliferation, survival, and cell cycle progression. Mutation of either K-RAS or BRAF is frequent in LGSC [28, 33]. Thus, two phase III trials, MILO and LOGS, have proven the great benefit of the MEK inhibitors *binimetinib* and *trametinib* as treatment for recurrent LGSC [34, 35]. Upcoming biomarker analyses should identify a subgroup of patients who selectively benefit from binimetinib [34]. To overcome resistance, treatments with either intra-pathway or inter-pathway combination are used [36]. Recently, the FDA has approved combinational treatment of *dabrafenib* (RAF inhibitor) and trametinib (MEK inhibitor) for all unresectable metastatic solid tumors with BRAF V600E mutation [37]. Based on this, combination of *vemurafenib* (BRAF inhibitor) and *cobimetinib* (MEK inhibitor) is currently investigated in the DETERMINE study for OC in case of BRAF V600 mutation [30].

Seventy percent of OC present mutations in PI3K/AKT/mTOR pathway. Hyperactive signaling either due to activating mutations in PI3K, AKT, and mTOR itself or due to loss of negative regulators as PTEN leads to constitutive activity of cell proliferation, motility, and survival [14, 28, 53]. High copy number variations of PIK3CA in 40% and mutation in 12% of OC, and encouraging results of a phase Ib trial, initiated the currently ongoing EPIK-O phase III study investigating *alpelisib* (PI3K inhibitor) and olaparib combination in platinum-resistant or -refractory recurrent OC (PRROC) without germline BRCAm (gBRCAm) [42]. Biomarker analyses will investigate the PI3K pathway, HRR status, and DNA damage/repair pathways to identify favored subgroups [42]. Besides, focal adhesion kinase (FAK) and its receptor anaplastic lymphoma kinase (ALK) are part of oncogenic signaling in OC. Whereas monotherapy with FAK inhibitor *defactinib* has not been successful, it is currently investigated in recurrent LGSC in combination with multikinase inhibitor *avutometinib* (RAMP301 trial) [17, 54]. It is designed as confirmatory trial aiming full approval by FDA. PROTAC (FAK proteolysis targeting chimeric molecule) degraders are promising in preclinical research for OC treatment [55]. Inhibition of Src, being part of the FAK signaling pathway, with *saracatinib* in a clinical study was not efficient [44].

Further interesting targets regarding cell proliferation have been the FRα and the insulin-like growth factor receptor (IGFR). Although IGF1R signaling is often dysregulated in OC, inhibition with monoclonal antibodies as *ganitumab* and kinase inhibitors as *linsitinib* have not been successful in clinical trials [56, 57]. This might be due to difficulties in maintaining insulin receptor signaling [17]. More than 90% of OC overexpress FRα, concomitant with especially increased JAK-STAT downstream signaling [58, 59]. Unfortunately, monoclonal antibodies as *farletuzumab* (MORAb-300) failed to show benefits in a phase III study in platinum-sensitive recurrent OC (PSROC) [39]. Nevertheless, FRα remains an interesting target for targeted transport systems. Disruption of AXL-axis in platinum-refractory women by *batiraxcept* also did not improve PFS [40].

Growing importance of biomarker-guided patient stratification is reflected in clinical trials as STAPOVER. Regardless of histological subtype and based on a signal transduction pathway assay, women with either estrogen receptor (ER), androgen receptor (AR), PI3K, or Hedgehog signaling pathway (HH) alterations are either treated with *letrozole* (aromatase inhibitor), *bicalutamide* (antiandrogen), or *itraconazole* (mTOR inhibitor) [43]. The expression of ERα is increased in ~ 80% of OC [60]. Clinical studies have proven that predominantly LGSC and endometrioid cancer show good responses to endocrine therapy [6, 61]. Ongoing MATAO study analyzes maintenance therapy with letrozole in OC [48]. Further studies include NRG-GY019 comparing letrozole monotherapy with carboplatin-paclitaxel followed by letrozole as maintenance therapy for LGSC [47].

### Inducing or accessing vasculature

**Fig. 2:**
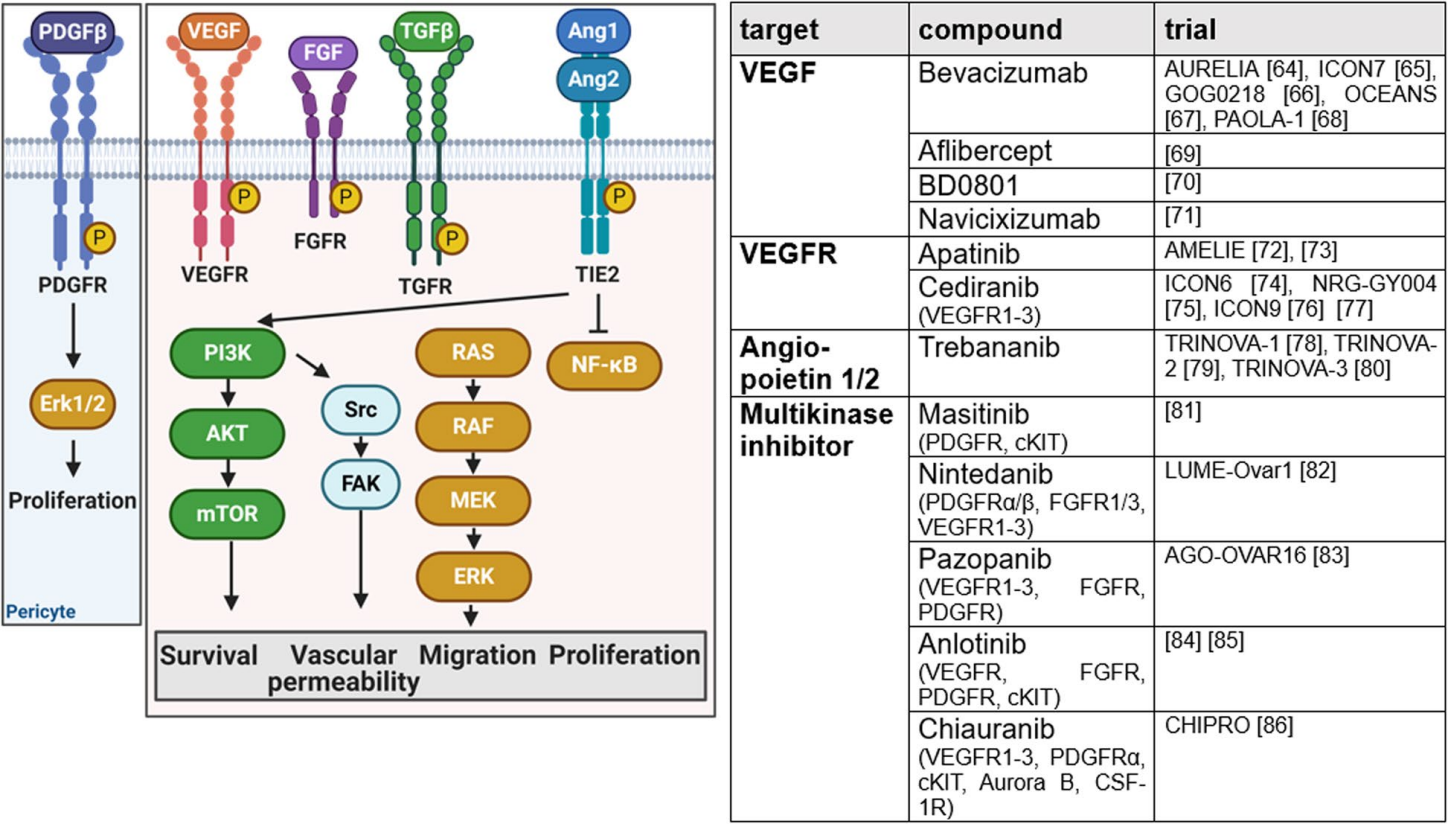
Inducing or accessing vasculature. In cancer, angiogenesis is stimulated by proangiogenic factors such as vascular endothelial growth factor (VEGF), tumor growth factor β (TGF β), and fibroblast growth factor (FGF), interacting with their receptors VEGFR, TGFR, and FGFR. Binding of angiopoietin to Tie2 receptor inhibits vascular maturation and platelet-derived growth factor (PDGF) promotes proliferation of pericytes. Increased activity of depictured signaling pathways leads to leaky neovasculature with high levels of apoptosis and excessive vessel branching. Currently ongoing and recently completed phase III trials targeting those pathways are listed in the table. This figure was created using Biorender.com [63–85]

To ensure sufficient nutrients, oxygen, and evacuation of waste or metabolites, endothelial cells are reactivated in cancer (Fig. 2). Angiogenesis stimulatory molecules, such as vascular endothelial growth factor A (VEGFA), tumor growth factor β (TGF β), and fibroblast growth factor (FGF), interact with its receptors VEGFR, TGFR, and FGFR [14, 62]. In contrast, by binding to the Tie receptor, angiopoietin (Ang) inhibits vasculature maturation [58]. Furthermore, platelet-derived growth factor β (PDGFβ) is involved in proangiogenic signaling, promoting proliferation of pericytes. Tumor neovasculature is characterized by leakiness, excessive vessel branching, and increased levels of apoptosis [14].

*Bevacizumab*, inhibitor of VEGF, was the first targeted and antiangiogenic therapy approved in front-line treatment and treatment of relapsed OC. Bevacizumab shows improvement in PFS but does not affect the OS rate. Clinically, it is of great value for patients with extensive ascites [63, 64]. Analyses based on the World Pharmacovigilance Database (FDA) and randomized controlled trials assess the long-term safety profile of bevacizumab as relatively positive [86]. Next to bevacizumab, several other VEGF-inhibitors have been investigated clinically. *Aflibercept*, a recombinant fusion protein trapping VEGF, was investigated in advanced chemoresistant OC with recurrent malignant ascites and led to less rapid ascites formation. Due to increased risk of fatal bowel perforation, aflibercept was not approved [68]. *Navicixizumab* is a fist-in-class bispecific antibody targeting delta-like ligand 4 (DLL4) and VEGF. Tumors responding to anti-VEGF therapy present low levels of DLL4, but unfortunately DLL4 is overexpressed in 72% of OC [87]. The high overall response rate (ORR) of 43.2% to navicixizumab in a phase Ib study in PRROC led to the initiation of a phase III trial with an estimated primary completion date in November 2023, which includes a further 12 months survival follow-up [88, 70].

*BD0801* is a monoclonal antibody blocking VEGF/VEGFR interaction, which is investigated in phase III trial with supposed primary completion in December 2023. Results are still pending [69]. Following the promising AEROC study, which investigated the VEGFR2 inhibitor *apatinib*, apatinib is further investigated in AMELIE trial for OC therapy and as maintenance therapy in combination with unapproved PARPi fluzoparib after first-line treatment [71, 72, 89]. Again, no results are available so far. *Trebananib* is a peptibody trapping Ang1/2. TRINOVA1-3 trials have investigated trebananib in combination with paclitaxel in ROC proving significant prolonging of PFS compared to placebo, in combination with pegylated liposomal doxorubicin (PLD) showing improved ORR but without PFS benefit and as first-line treatment in combination with carboplatin and paclitaxel unfortunately demonstrating only minimal benefits for patients [77–79]. With the shift in patient stratification from recurrent epithelial OC (TRINOVA1-2) to epithelial OC, primary peritoneal or fallopian tube carcinoma (TRINOVA 3), the benefit has decreased.

Several multikinase inhibitors are part of treatment strategy investigations for OC. *Cediranib* is a multikinase inhibitor targeting VEGFR1-3. ICON6 and NRG-GY004 study failed to show significant OS benefits of cediranib given concurrently to standard of care therapy, given as maintenance therapy and given in combination with olaparib [73, 74]. However, worthwhile activity was suspected, featuring ICON9 study, which investigates maintenance therapy of olaparib with cediranib or placebo. Results are expected for 2025 [75]. Another study (phase II/III) evaluating cediranib and olaparib combination for recurrent or metastatic OC, is ongoing [76]. Likewise, no results of *masitinib*, a multikinase inhibitor recently approved for amyotrophic lateral sclerosis, in combination with gemcitabine in PSROC have been published so far [80]. Multikinase inhibitor *nintedanib*, investigated in 2009 in combination with carboplatin and paclitaxel, has not been approved due to absence of OS benefits although realizing PFS benefits [81]. Sadly, AGO-OVAR16 study, which investigated the multikinase inhibitor *pazopanib* (Votrient), did not confirm suspected OS benefit of MITO11 study [82, 90]. Furthermore, the combination of TQB2450, a programmed death-ligand inhibitor, and *anlotinib* is currently investigated [83, 91]. Anlotinib is further investigated in a phase I/IIa/III study in ROC concurrently to standard of care and as maintenance therapy [84]. CHIPRO is an ongoing phase III trial investigating the multikinase inhibitor *chiauranib* targeting VEGFR1-3, PDGFRα, cKIT, Aurora B, and CSF-1R, in combination with weekly paclitaxel in patient with PRROC [85].

### Evading growth suppressors

**Fig. 3:**
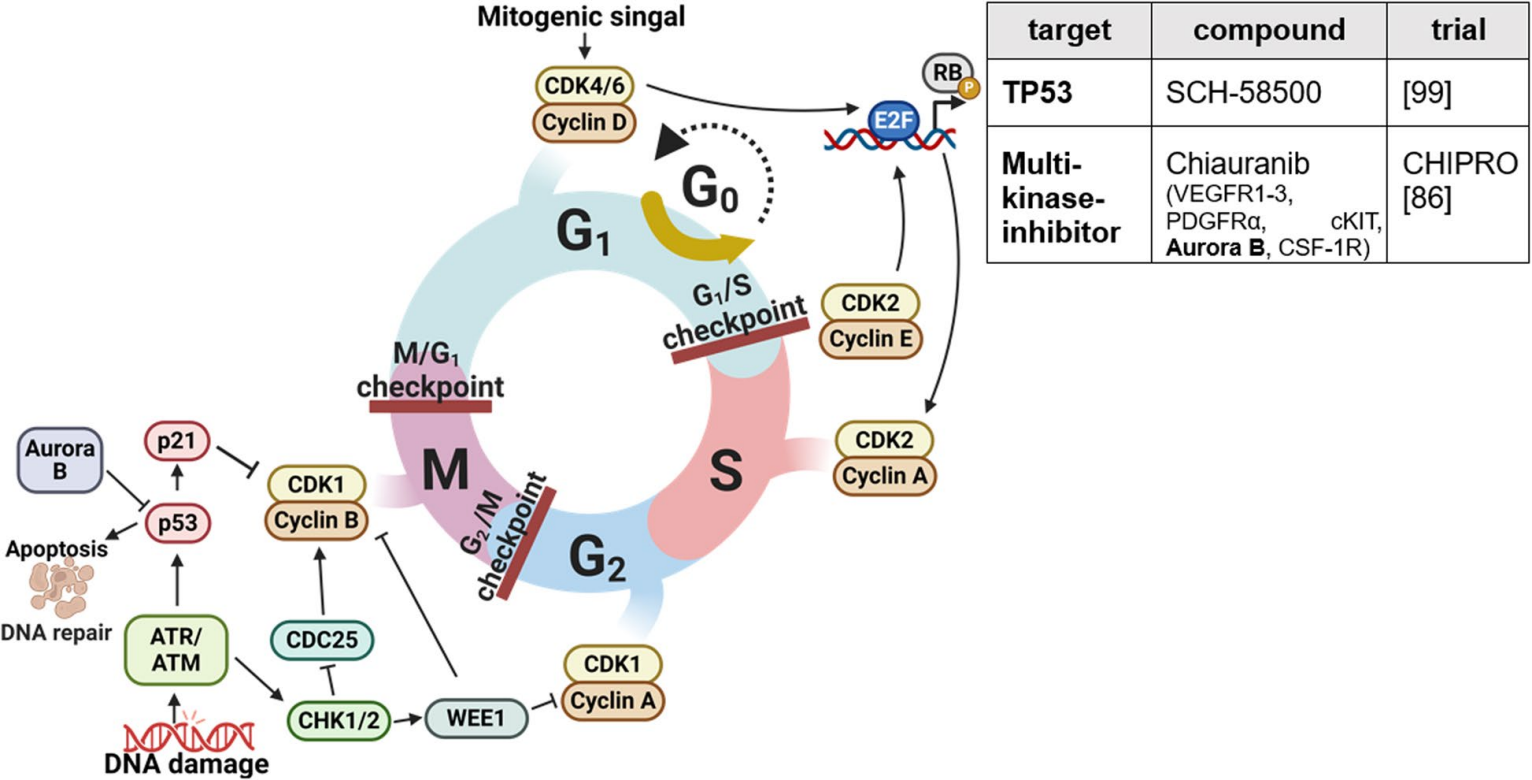
Evading growth suppressors. Cell cycle progression is strictly regulated by checkpoints controlled by cyclin-dependent kinases (CDKs)/cyclin-complexes. Due to frequent aberrations in checkpoint control, as well as alterations in critical gatekeepers, uncontrolled cell cycle progression is a common feature in OC. Current strategies in phase III studies targeting evasion of growth suppressors are listed in the table. This figure was created using Biorender.com [85, 98]

Processes that circumvent growth-inhibiting signals are a main characteristic of cancer cells. Absence of critical gatekeeper of cell cycle proliferation, in particular p53 (tumor protein p53) and RB (retinoblastoma-associated), leads to uncontrolled cell growth. RB mainly regulates extracellular signals; TP53 mostly processes intracellular signals and can induce cell cycle arrest to repair DNA damage or start apoptosis [14]. Both tumor suppressors are commonly altered in OC (e.g., TP53 in over 90%) [92]. Cell cycle progression is tightly regulated by cyclin-dependent kinases (CDKs) interacting with cyclins (Fig. 3). The frequent dysregulation in cancer makes them promising targets for therapy.

G1-S-phase transition is controlled by cyclin D/CDK4/6 and cyclin E/CDK2 complexes. A positive feedback loop, wherein mitogenic stimuli such as c-myc increase cyclin D expression which inactivates together with CDK4/6 RB and results in release of transcription factor E2F, leads to G1/S-transition and cyclin E expression, which can further promote its own expression independent of other stimuli [93]. G2/M transition is regulated by CDK1/2 building a complex with cyclin A/B, which formation is under control of DNA damage response ATM-CHK2 and ATR-CHK1 axis. Activated ATR causes phosphorylation of CHK1, which leads to reduced CDK activity through proteasomal degradation of CDC25 and causes a delay in the cell cycle [94, 95]. Further, Wee1 kinase is activated by CHK1, subsequently inhibiting CDK1/2. Activated ATM/ATR further activates p53-signaling, among others leading to upregulation of p21, and thereby stabilizes the RB-E2F complexes and prevents apoptosis [96]. To support cell cycle progression after DNA repair, Aurora kinase A activates polo-like kinase 1 (PLK1), which in turn inhibits Wee1 activity, whereas Aurora kinase B accelerates degradation of p53. Defects in G1/2 transition are common in cancer cells, making them more reliant on intra S and G2/M checkpoints for survival [95, 97].

CCNE1 (cyclin E1) amplification is a common copy number variation (> 20%) in HGSC. CCNE1 amplification leads to facilitated cell cycle progression and replicational stress accompanied by genomic instability [6]. Thus, treatment strategies focus on CDK inhibitors, inhibition of ATR/CHK1/WEE1 axis and restoration of p53.

Preclinical studies have demonstrated a benefit of CDK4/6 inhibitors, such as *palbociclib* and *ribociclib*, already approved for breast cancer, in estrogen receptor-positive cancer. A clinical phase II study investigating ribociclib in combination with letrozole in ROC has proven high response rates in LGSC [99]. Recently, the ALEPRO study started investigating another CDK4/6i, *abemaciclib*, together with letrozole in patients with estrogen receptor-positive rare OCs as an international, multicentre, open-label, single-arm phase II study [100]. Patient-derived organoids (PDOs) have responded well to *flavopiridol*, a multiple CDKi, which was clinically confirmed (phase II) in cisplatin-resistant recurrent OC [17, 101, 102].

Inhibition of ATR/CHK1/WEE1 axis enhances sensitivity of cancer cells to treatment due to uncontrolled cell cycle progression and high replicational stress, therefore being interesting for combinational treatments [6, 95, 103, 104]. *Ceralasertib*, an ATR inhibitor, combined with PARPi has proven a clinical benefit rate of 62.5% in HRD and/or BRCAm PARPi-resistant ROC [105]. ATR inhibitor *prexasertib* was granted FDA Fast Track designation due to promising interim phase II study results [106, 107]. Unfortunately, other CHK inhibitors caused severe side effects [17]. Wee1, upregulated in OC, can be inhibited by *adavosertib*, thereby increasing sensitivity towards chemotherapy in TP53 mutant HGSC [108]. Inhibition of cell cycle progression by Aurora kinase A inhibitor, *alisertib*, and the PLK1 inhibitor, *volasertib*, has shown in phase II studies to be beneficial and merit further investigation [109, 110].

Apart from this, restoration of p53 is a promising anticancer approach. Gene therapy with recombinant adenovirus p53 (*SCH-58500*) had been shown to be safe and favorable in phase I/II trials and progressed to a phase II/III trial in 1999. However, results have not been published. Further efforts aim to re-engineer p53, with adenoviruses or nanoparticles as carrier systems (e.g., *Au-C225*). Peptide-based p53 therapy, such as the *p53-SLP* vaccine, failed to show benefits in phase II trial. Promising small molecules reactivating mutant p53 are *APR-246* and *zinc metallochaperones* [111]. HSP90i, *ganetespib*, which promotes degradation of mutant p53 by MDM2 machinery, represents another approach [20]. In addition, the degradation of p53 can be influenced by the multikinase inhibitor *chiauranib*, which inhibits AURORA kinase B and is being investigated in the ongoing CHIPRO study together with paclitaxel [85].

### Resisting cell death

**Fig. 4:**
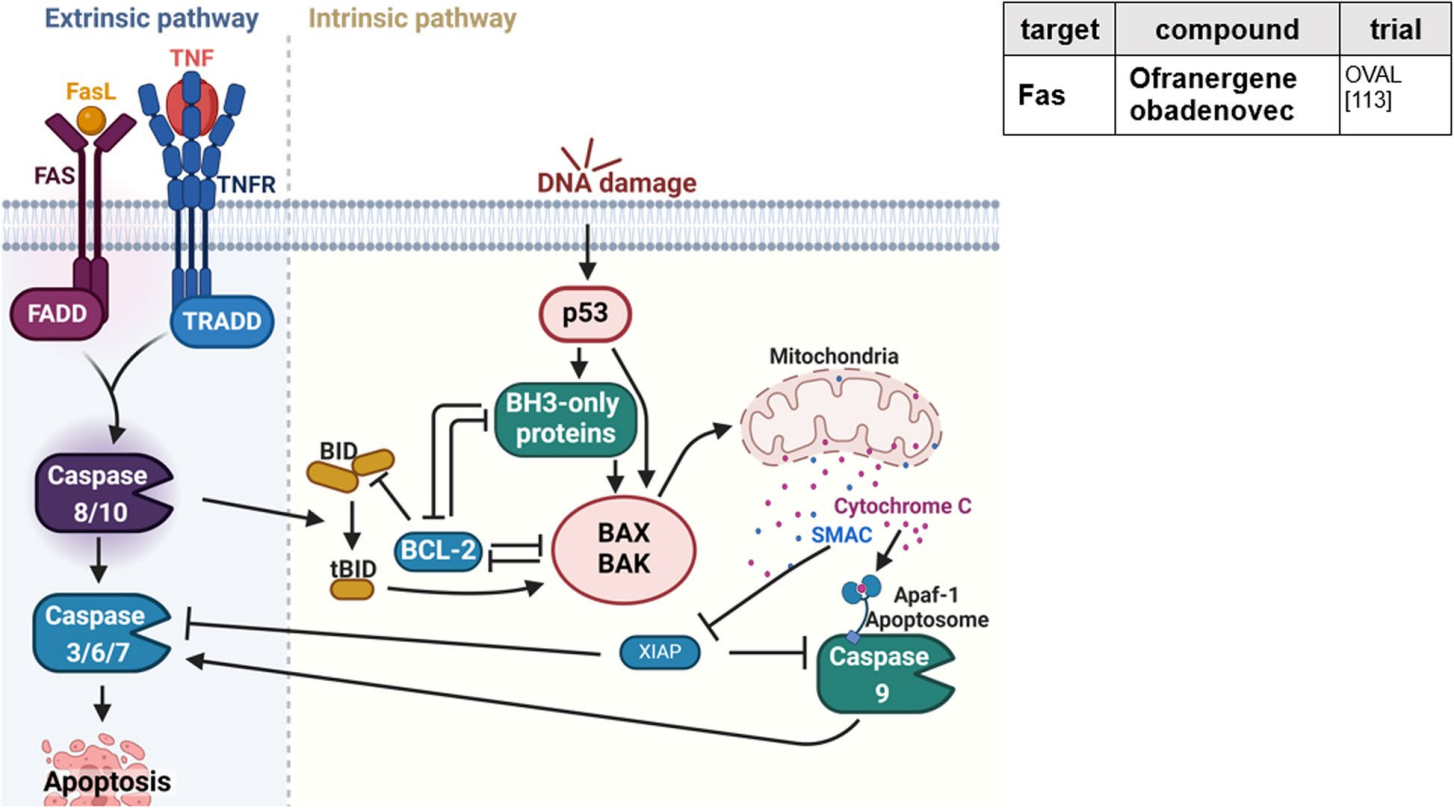
Resisting cell death. With regard to apoptosis induction, extrinsic and intrinsic pathways are differentiated. Several mechanisms of cancer cells are known to circumvent apoptosis. Treatment strategies to inhibit resistance to cell death are listed in the table. TNF, tumor necrosis factor; TRADD, TNFR1-associated death domain protein; FADD, Fas-associating protein with death domain. This figure was created using Biorender.com [112]

Apoptosis is a pathway to eliminate cells harboring mutations (Fig. 4) [14]. In the extrinsic pathway, Fas-ligand-receptor interaction activates caspase 8 and, in the following, stimulates effector caspases triggering apoptosis. The intrinsic pathway is activated consequently to DNA damage and excessive oncogenic signaling. While regulators such as BCL-2 inhibit proapoptotic proteins as BAX and BAK, p53 promotes apoptosis by upregulation of BH-3-only proteins Noxa and Puma, which in contrast activate BAX and BAK. Subsequently, release of cytochrome c out of the outer mitochondrial membrane is promoted, featuring activation of caspase cascades. Cleavage of Bid to tBid displays cross activation of intrinsic pathway in case of extrinsic induced apoptosis. Inhibitors of apoptosis (IAP), e.g., XIAP and survivin, are important regulators of apoptosis, which suppress activity of intrinsic and extrinsic pathway by caspase inhibition [113].

OC is known for increased expression of antiapoptotic signals and survival signals, as well as decreased expression of proapoptotic signals [4, 14, 21]. Thus, these represent valuable targets for therapy. BH3 mimetics, such as *ABT-737* and *WEHI-539*, which antagonize BCL-XL, have shown to synergize with carboplatin in cell growth assays, as does *ABT-263* (navitoclax) with PARPi in vitro [114, 115]. Clinically, monotherapy of navitoclax, investigated in a phase II trial, was only marginally effective [116]. Another approach seems to be upregulation of BH3-only proteins with *naftopidil* [21].

*Ofranergene obadenovec* (VB-111) is a viral-based therapy, delivering a Fas-TNFR1 chimeric pro-apoptotic protein. It is supposed to drive endothelium specific expression and induction of apoptosis, leading to vascular disruption and activation of immune system. A phase III trial completed in July 2022 investigated VB-111 in combination with paclitaxel. Further analyses included subgroup analyses, quality of life, histopathology, and biomarkers. No improvement in PFS or OS was observed for PRROC [112, 117]. 

### Avoiding immune destruction

**Fig. 5:**
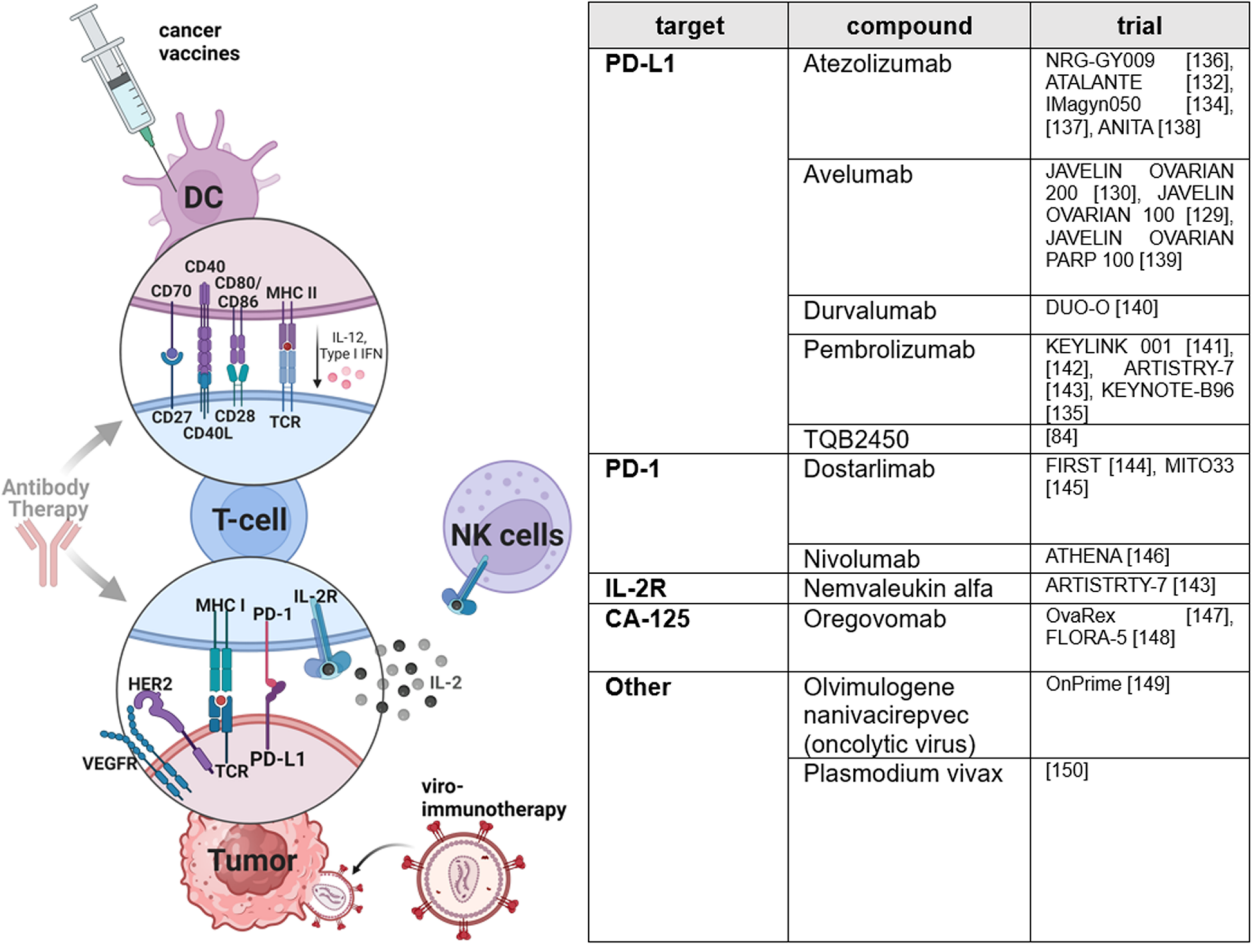
Avoiding immune destruction. Expression of immune checkpoint molecules and programmed cell death ligands compromise immunostimulatory interaction of tumor, T, and dendritic cells. In addition, OC is characterized by highly immunosuppressive tumor microenvironment, which further attenuates antitumoral immune response by secretion of cyto- and chemokines. Current endeavors fighting immune evasion encompass immune checkpoint inhibitors, cancer vaccines, viro-immunotherapy, and interleukin application. Recently completed and currently ongoing phase III trials targeting immune evasion of OC are listed in the table. This figure was created using Biorender.com [83, 128, 129, 131, 133–149]

Since the approval of *ipilimumab* in 2011, immune checkpoint inhibitors (ICI) have revolutionized the treatment of many solid cancer types, except for OC [118]. Long-term follow-ups (≥ 3 years) of patients treated with ipilimumab indicate a consistent quality of life and an improvement in OS [119]. ICI prevent interaction of receptor and corresponding ligands such as CTLA-4/CD80/86, PD-1/PD-L1/2, PD-L1/2/CD80, and LAG-3/MHC-II, and thus override tumor’s survival mechanisms, especially the inhibition of T cell activity (Fig. 5) [18, 120].

For single-agent therapy with ICI, such as anti-PD-L1 *pembrolizumab* and anti-PD-1 *nivolumab*, accordingly to studies as KEYNOTE-100 and NINJA, limited efficacy had been announced and no biomarkers have been identified [121, 122]. Only for clear cell carcinoma partial responses were detected. Causes of failure to improve patients’ outcome include the comparatively poorer ability of the immune system to show antitumoral response. Efficacy of ICI depends on high PD-L1 expression, low prevalence of somatic copy number variations, and the immunosuppressive properties of the TME [6, 17]. Although BRCA-1/2 deficient cells show the highest PD-L1 expressions and immunogenicity due to mutational burden, therapy response to ICI was not major in BRCA-1/2-deficient cells compared to BRCA-1/2-proficient cells [17, 122, 123]. TME of OC is highly immunosuppressive and contains various types of immune cells. High amount of tumor-infiltrating lymphocytes (TIL) as well as high ratio of CD8 + TILs/CD4 + regulatory T cells (T_reg_) have established positive prognostic properties in OC [124–126]. Due to changed expression of surface molecules and secretion of immunosuppressive chemokines and cytokines by tumor cells and innate immune cells, as myeloid-derived suppressor cells, immature dendritic cells, and tumor-associated macrophages of M2 phenotype, immunosuppressive T_regs_ get activated and effector T cells and natural killer (NK) cells get inhibited [6, 124]. Non-immune cell TME is mainly built by cancer-associated fibroblasts, cancer-associated adipocytes, endothelial cells, and pericytes and supports immunosuppression by growth factors and cytokine production [18, 124, 127]. Increasing knowledge about functions of TME exhibits further strategies to improve treatment. With identification of predictive biomarkers, immunotherapy can either target inhibition of immunosuppression or support stimulation of the immune system.

In view of the complexity of the mechanisms, it seems sensible to address different signaling pathways by combination treatment. Since combination of ICI *avelumab* and standard of care treatment (carboplatin-paclitaxel) likewise did not prove benefits, nor in first-line, nor in second-line (JAVELIN 100/200), and also the combination of PLD with ICI pembrolizumab or *durvalumab* did not improve efficacy significantly despite a pro-antigen-presenting effect, current research focuses on combination with other agents as PARPi and bevacizumab [128–130]. In the ATALANTE-trial, which investigated *atezolizumab* in combination with* bevacizumab* and chemotherapy in ROC, the coprimary PFS in intention to treat population and PD-L1 positive populations was not reached [131]. AGO-OVAR 2.29/ENGOT-ov34 study results, recently presented at the ASCO Annual Meeting 2024, similarly did not show OS or PFS benefits for atezolizumab combined with bevacizumab in non-platinum-based chemotherapy in ROC compared to placebo [132]. Likewise, IMagyn050 study did not verify a PFS benefit in newly diagnosed OC [133]. Results of NRG-GY009 study, investigating atezolizumab-PLD-bevacizumab combinations in ROC, are still pending, and KEYNOTE-B96, investigating pembrolizumab in addition to weekly paclitaxel with or without bevacizumab in PRROC, is ongoing [134, 135].

Preclinically determined immunomodulatory properties of PARPi, such as increased neoantigen formation, increased PD-L1 expression and increased immune cell infiltration, built rationale to combine them with ICI [150]. Following phase I/II studies proving benefit of ICI-PARPi combinations regardless of BRCA-/HRD/PD-L1 status and providing proof of advanced OS rates by therapy triplet including bevacizumab, several phase III studies are currently ongoing [151, 152]. JAVELIN Ovarian PARP 100 trial evaluated treatment efficacy of *avelumab* in combination with chemotherapy followed by maintenance therapy with avelumab and *talazoparib* in advanced OC but was stopped in 2019 due to missing benefits of avelumab for unselected patients in front-line setting observed in interim-analysis of JAVELIN Ovarian 100 trial [153]. The results of the ANITA study, which investigated application of chemotherapy in combination with *atezolizumab *and niraparib in patients with ROC, have been recently presented at the ESMO Annual Congress 2023. They indicate a PFS benefit for only non-BRCAm OC [137]. Large ongoing studies further investigating PARPi-ICI combination in first-line and recurrent situations include KEYLINK-001, FIRST, MITO-33, and the COMBO arm within ATHENA-trial [140, 143–145].

The DUO-O study investigated the benefits of durvalumab therapy in combination with chemotherapy and bevacizumab, followed by maintenance therapy with *durvalumab* (anti-PD-L1),* bevacizumab*, and olaparib, in newly diagnosed advanced OC without BRCA mutation. For the first time, benefits of PARPi and ICI were seen. Durvalumab and olaparib combination led to a significant improvement in PFS, from 19.3 months to 24.2 months (HR 0.63). Considering only HRD-positive patients, a median PFS of 37.3 months (vs. 23 months) was reached (HR 0.49) [139]. This gives rise to hope for further advances in ICI-PARPi-bevacizumab combination. Nevertheless, critics fault the lack of an olaparib maintenance control arm in DUO-O study. Follow-up studies will provide further insights into the long-term benefits. So far, durvalumab is not approved in OC.

Other combinational strategies include testing *TQB2450* (PD-L1 inhibitor) in combination with anlotinib [83]. Backed on preclinical and phase Ib data for PRROC showing ORR of 47.1%, a phase III trial is currently conducted in China [91]. *Nemvaleukin alfa*, a novel engineered IL-2 cytokine fusion protein, is presently investigated in ARTISTRY-7 trial in combination with ICI pembrolizumab in PRROC and has already gained fast track designation by FDA [142]. Due to sterical occlusion, it only stimulates IL-2 receptors (IL2-R) and activity of T_Eff_ and NK-cells and not those of T_regs_, thereby preventing capillary leak syndromes as often noticed in case of simple IL-2 administration [142, 154]. ARTISTRY-1 trial provided evidence for the activity and safety of nemvaleukin alfa in PRROC [155].

Cancer vaccines are a growing field of research in OC. Peptide-based vaccines consist of known or predicted tumor-associated antigens (TAAs) administered with adjuvants to enhance immunogenicity. Presentation of processed antigens by antigen presenting cells and dendritic cells (DC) leads to activation of T_Eff_ cells and cytotoxicity by B cells [156]. Common TAAs to target in OC are FRα, HER2, CA125 (MUC16), MAGE-A4, NY-ESO 1, and mesothelin [4, 6, 18]. *Oregovomab*, a CA-125-specific murine monoclonal antibody, is already investigated as cancer vaccine in phase III trials since 2002. Despite missing improvement of clinical outcome using oregovomab as maintenance therapy in advanced OC, it is currently investigated as front-line therapy in newly diagnosed advanced epithelial OC in combination with paclitaxel and carboplatin in FLORA-5 study [146, 147].

Oncolytic viruses (OV) act by direct oncolysis of infected cells and contribute to indirect activation of the host immune system due to release of danger associated molecular patterns, viral antigens, and TAAs [18, 157]. Genetic engineering enables expression of transgenes, increases tumor specificity, and grows oncolytic potency [156]. OV therapy also affects the TME [18]. Following the success in phase II trial, *GL-ONC1* (olvimulogenic nanivacirepvec) is currently investigated in the phase III OnPrime trial as front-line treatment in combination with platinum-based chemotherapy with or without bevacizumab [148, 158]. Further approaches but still in preclinical research include infected cell vaccines (ICVs), considering autologous tumor cells as vehicles to tumor niche, thereby turning immunologically “cold” tumors into “hot” tumors [4, 18].

Another line of attack is adoptive cell therapy (ACT), a transfer of autologous or allogeneic immune cells. Besides successful use of TIL for ACT after platinum-based chemotherapy in 1995, the utilization of dendritic cell vaccines (DCV) pulsed with TAAs is likewise interesting [159, 160]. Also, chimeric antigen receptor T cells (*CAR-T*) are actively investigated in OC, allowing an antigen-specific recognition of cancer cells and major histocompatibility complex (MHC)-independent activation of T cells [161]. However, CAR-T cell therapy still faces many issues, as off-target effects and tumor heterogeneity [162]. Bispecific antibodies (e.g., *ubamatamab* and *REGN5668*), which activate the T cell response by simultaneous binding to tumor and T cells, are currently in phase I/II trials [163–165]. In addition, in 2024, an unconventional phase II/III study is expected to test the effect of *Plasmodium vivax* on OC [149].

### Genome instability and mutation

Germline, somatic, and epigenetic mutations compromising DNA damage-detection and -repair lead to genome instability, a fundamental feature of cancer [14]. Genome instability is associated with deficiency in homologous recombination (HR), which is present in 41–50% of OC and is utilized by therapies targeting DNA repair [181]. In addition to BRCA1 and BRCA2 mutations, various other genetic mutations and amplifications, e.g., in RAD51C, ATM/ATR, PTEN and CHEK2, have an impact on HRD in OC [92]. Since recent clinical research has proven predictive potential of HRD regarding response to platinum-based and PARPi therapy, HRD tests were introduced to diagnostic algorithm of OC [182, 183]. Germline and somatic mutations are screened by next generation sequencing [181]. Further HRD tests focus on identification of loss of heterozygosity, telomeric allelic imbalances, and large-scale transitions, the “scars” of genomic instability [184]. So far, two commercially FDA approved tests are available: FoundationOne by Foundation Medicine and myChoice HRD test by Myriad Genetics. Since mutagenesis during tumor evolution can compromise accuracy of HRD tests, much effort is put into development of functional HRD assays, as quantification of nuclear RAD51, to display current HRD status [184].

**Fig. 6:**
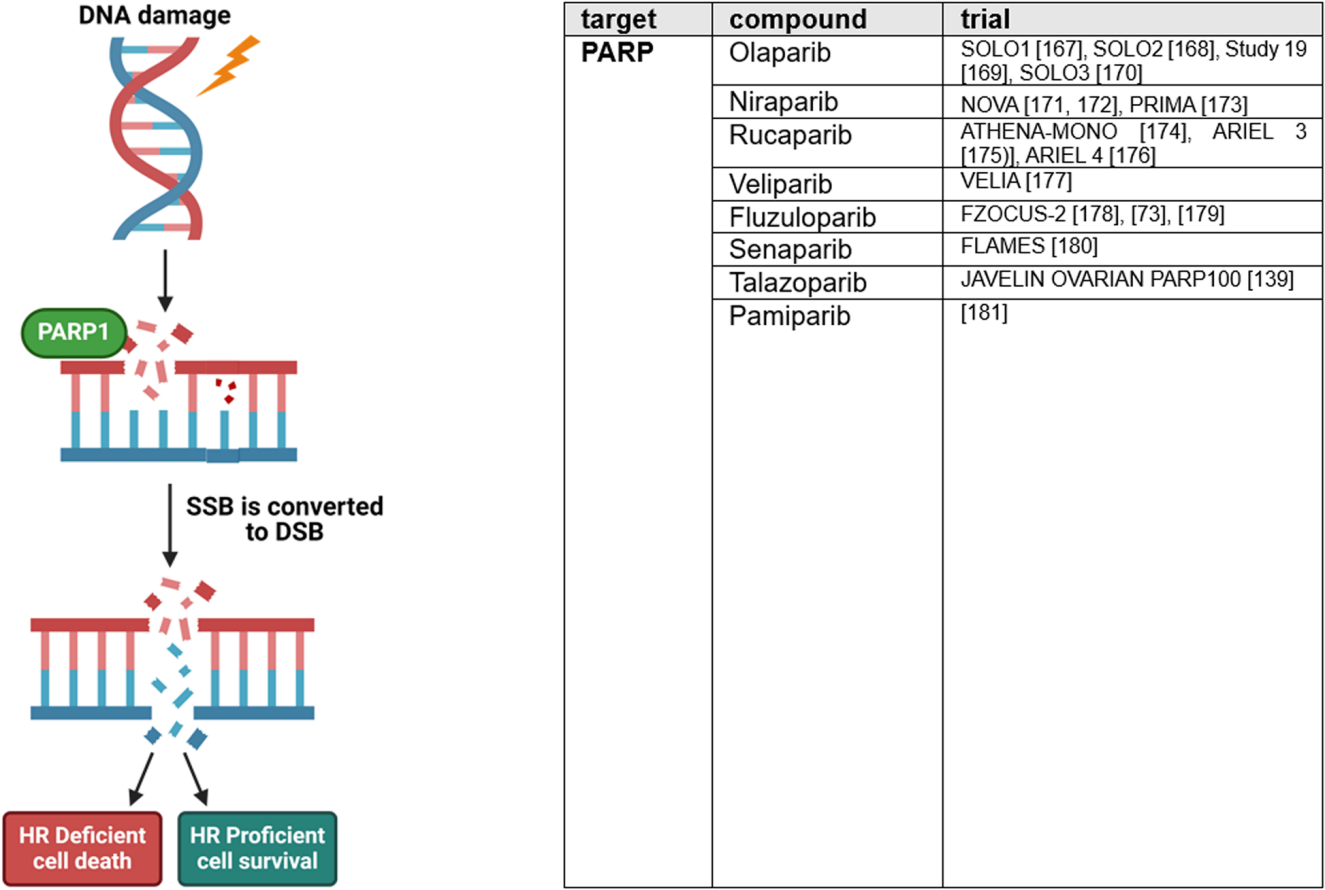
Genome instability and mutation. High quality repair of DNA damage is crucial to maintain genomic stability. In case of DNA damage repair defects, as homologous repair deficiency or artificially induced defects by PARP inhibition, DNA damage leads to genomic instability or cell death due to synthetic lethality. Recently completed and currently ongoing phase III trials targeting genome instability of OC are listed in the table. SSB, single-strand breaks; DSB, double-strand breaks. This figure was created using Biorender.com [72, 138, 166–180]

Based on synthetic lethality, PARPi are highly efficient in OC (Fig. 6). Among others, PARPi inhibit repair of DNA single-strand breaks and thereby cause accumulation of DNA double-strand breaks. Deficiency of high-quality HR and concurrent inhibition of alternative end joining (alt-EJ) by PARPi, as well as dependency on more error-prone non-homologous end joining (NHEJ) to repair DSBs, leads to accumulation of mutations, unregulated cell division, and apoptosis [95, 181, 184, 185].

Thus, today PARPi are considered as first-line standard of care maintenance therapy of OC after response to platinum-based therapy. Based on several phase III studies, investigating *olaparib*, *niraparib*, and *rucaparib* in first-line setting, including SOLO-1, PRIMA, and ATHENA trial which have been reviewed in detail elsewhere, olaparib is approved for first-line maintenance treatment of advanced OC with BRCAm; niraparib is approved regardless of HRD status [186, 187]. Olaparib approval was extended by its use in combination with bevacizumab as first-line maintenance therapy in HRD positive OC due to PAOLA-1 study [186].

Based on SOLO2 and study 19, olaparib is also approved as maintenance treatment for ROC [188]. Caused by new results of ARIEL-3 and NOVA study, approval of rucaparib and niraparib has recently been restricted to tumors with BRCAm in recurrent situations [10,108,171,190-195].

PARPi as monotherapy in late line treatment have been discouraging so far. In SOLO 3 study, which treated gBRCA1/2 mutated HGSC PSROC with olaparib monotherapy, no significant difference in OS and PFS2 compared to placebo group was seen [195, 196]. Likewise, third-line monotherapy with rucaparib for BRCAm OC was withdrawn in June 2022. New OS results of ARIEL 4 study, contrary to initially encouraging PFS results, favored chemotherapy over rucaparib [175, 196]. QUADRA study, a single-arm phase II study, investigated niraparib as fourth line or later treatment in HRD positive ROC. In September 2022, approval for this indication was voluntarily retrieved [197, 198]. Based on this long-term follow-up, the FDA withdrew approval for the PARP inhibitors olaparib, rucaparib, and niraparib for single-agent treatment. Long-term profile (> 2 years) proved olaparib to be safe and well tolerated [168].

Other PARPi, as for example *veliparib*, did not reach clinical approval despite phase III study VELIA proving a longer PFS compared to carboplatin-paclitaxel alone [176]. New PARPi as *fluzuloparib* and *pamiparib* are currently evaluated in clinical trials in China [72, 177]. In China, fluzuloparib is already approved for treatment of gBRCAm PSROC since 2020 [199]. The new PARPi *senaparib* also offers promising PFS benefits as maintenance therapy in first-line treatment, regardless of biomarkers [179]. However, PARPi treatment is associated with increased risk of myeloid-neoplasia, due to PARP2 inhibition [6]. Therefore, selective inhibition of PARP1 becomes a new strategy and promising agents as the selective PARP1 inhibitor *AZD5305* are already under clinical evaluation [200, 201].

Since PARPi are chemosensitizing, they are popular agents for combination studies [202]. Combinational designs can be useful to overcome resistance mechanisms to PARPi and platinum-based therapy, including restoration of HR, upregulation of multidrug-resistance channels, or replication fork stabilization [4, 200, 203, 204]. Furthermore, cytotoxic effects of chemotherapeutics can be enhanced by combination with PARPi, be it through accumulation of topo I-DNA complexes or by induction of replication stress thereby sensitizing to cell cycle checkpoint inhibitors (ATRi/CHKi/WEE1i) [205].

Next to high sensitivity towards PARPi, genome instability in OC offers a broad range of targets to inhibit. Due to common deficiencies in HR, OC are more reliant on alternative repair mechanisms as NHEJ and alt-EJ [206]. Inhibition of alt-EJ regulating Pol-θ with agents as *novobiocin* and *ART558*, as well as inhibition of NHEJ regulating DNA-dependent protein-kinase catalytic subunits via *peposertib*, is already under early clinical investigation [6, 107, 207–209].

Other strategies targeting genome instability, include stabilization of G-quadruplex structures, for example by *pidnarulex* [204, 210]. Ubiquitin-specific protease 1 (USP-1) inhibitors as *KSQ-4279* promote degradation of DNA repair proteins and are already part of clinical studies [201, 211]. Also interesting is *AsiDNA™*, which mimics DNA double-strand breaks and subsequently induces apoptosis of cancer cells [107, 212].

### Tissue invasion and metastasis

Invasion and metastasis formation builds another basis of cancer progressing to higher malignancy and comprises cell detachment, dissemination, and implantation [124]. Epithelial-mesenchymal transition (EMT) represents a comprehensive model, how cancer cells acquire ability to detach from primary tumor and increase migratory capacity [4, 14]. Contrary to other epithelial cancers, EMT seems to be subsidiary for metastasis formation in OC, being more reliant on passive exfoliation of tumor cells by fluid current to peritoneal cavity [20, 124, 213]. Several mechanisms are described to overcome anoikis, a specific form of apoptosis usually induced upon loss of cell–matrix contact, including FAK activation and overexpression of RAB25, BCL-2 family proteins, and EGFR [124]. Ascites, with its unique TME, promotes cell metastasis. Surface markers expressed on OC as MUC16/CA125 and mesothelin, support adhesion to mesothelial cells [124].

However, typical changes associated with EMT, such as low expression of E-cadherin, are correlated with a poor outcome, making it an interesting target for OC treatment [214]. EMT is driven by transcription factors (EMT-TF) such as Slug, Snail, Zeb-1, and Twist, which increase expression of mesenchymal adhesive and cytoskeletal proteins (N-cadherin, Vimentin, Fibronectin, ß1-ß3-integrins, matrix metalloproteinases) and decrease epithelial state proteins (occludin, claudin, α6ß4 integrins, cytokeratin) [215]. Thus, current research focuses on inhibition of abovementioned features enabling invasion and metastasis and targeting signaling pathways that activate EMT-TF expression, including TGF ß signaling, Wnt pathway and mitogenic growth factor receptors triggering PI3K-AKT, RAS/RAF/MAPK, p38MAPK, and JNK pathways resulting in NFkB expression [215].

*FANG vaccine* (gemogenovatucel-T, vigil) is a tumor cell vaccine that stimulates dendritic cells and promotes downregulation of TGF ß1/ß2 [216]. TGF ß has multiple functions. It increases EMT-TF expression via SMAD, ERK, and PI3K/AKT signaling [215, 217]. Besides, TGF ß acts strongly immunosuppressive by inhibition of antitumoral T cell responses [218]. In phase II studies, the vaccine prolonged the time to recurrence in the first-line treatment of epithelial OC after standard therapy [219]. The VITAL study (IIb) analyzed vigil in stage IIIb-IV OC after complete clinical response to debulking surgery and primary chemotherapy [220]. Despite good toleration of treatment, primary endpoint was not reached [216]. This highlights the importance of both efficacy and safety profiles. Subgroup analysis has proven OS benefit for HR proficient women (HR 0.342) [221].

**Fig. 7:**
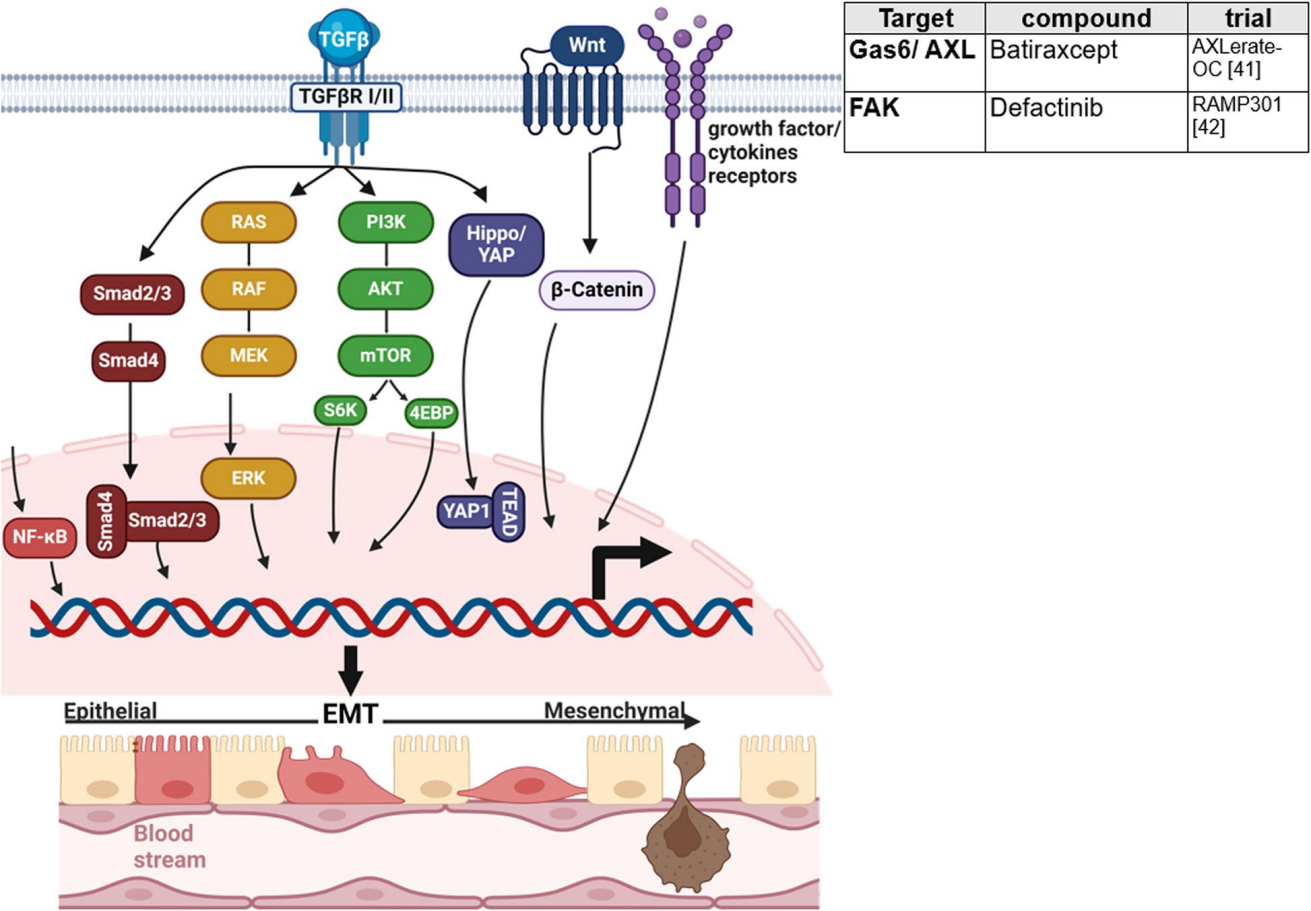
Tissue invasion and metastasis. Tissue invasion and metastasis are complex processes, modulated by various signaling pathways. Epithelial-mesenchymal transition and expression of its regulating transcription factors, which are controlled by TGF β, Wnt, and growth factor signaling, is pivotal. Recently completed and currently ongoing phase III studies targeting tissue invasion and metastasis are listed in the table. EMT, epithelial-mesenchymal transition. This figure was created using Biorender.com [40, 41]

MET tyrosine kinase receptor and its ligand hepatocyte growth factor are involved in EMT activation by upregulation of SNAIL [215]. *Cabozantinib* is a multikinase inhibitor targeting Met, VEGFR2, Ret, Flt3, Kit, and Tie 2 and has been investigated in two phase II trials in ROC [222, 223]. Unfortunately, monotherapy failed to reach good response [224, 225]. Ongoing studies focus on application in germ line cell tumors and in combination with atezolizumab [226, 227]. Since FAK and AXL are also involved in metastasis formation, *defactinib* (FAKi) and *batiraxcept* (AXL decoy protein) are currently investigated in ROC phase III trials (Fig. 7) [40, 41, 228, 229].

Downstream activation of NFκB, e.g., by. PI3K-AKT signaling promotes inflammation and invasion among others by expression of MMPs [230]. *Belinostat*, a histone deacetylase complex (HDAC) inhibitor, reduces NFκB gene transcription by hypoacetylation. Despite initial encouraging results of a phase Ib/II study proving an ORR of 43% in ROC to treatment with belinostat, carboplatin, and paclitaxel, a phase II clinical trial in PRROC had to be stopped due to lack of drug activity [231–233].

*Catumaxomab*, approved in 2009 but withdrawn in 2014 due to insolvency, is a trifunctional bispecific antibody, targeting EpCAM [234]. Phase II/III trials, investigating catumaxomab and paracentesis, have shown slight improvement in puncture-free survival [235, 236]. Since August 2022, catumaxomab is once again under evaluation by CHMP (Committee for Medicinal Products for Human Use, EMA) for approval [237].

### Further hallmarks to target/perspectives

**Fig. 8:**
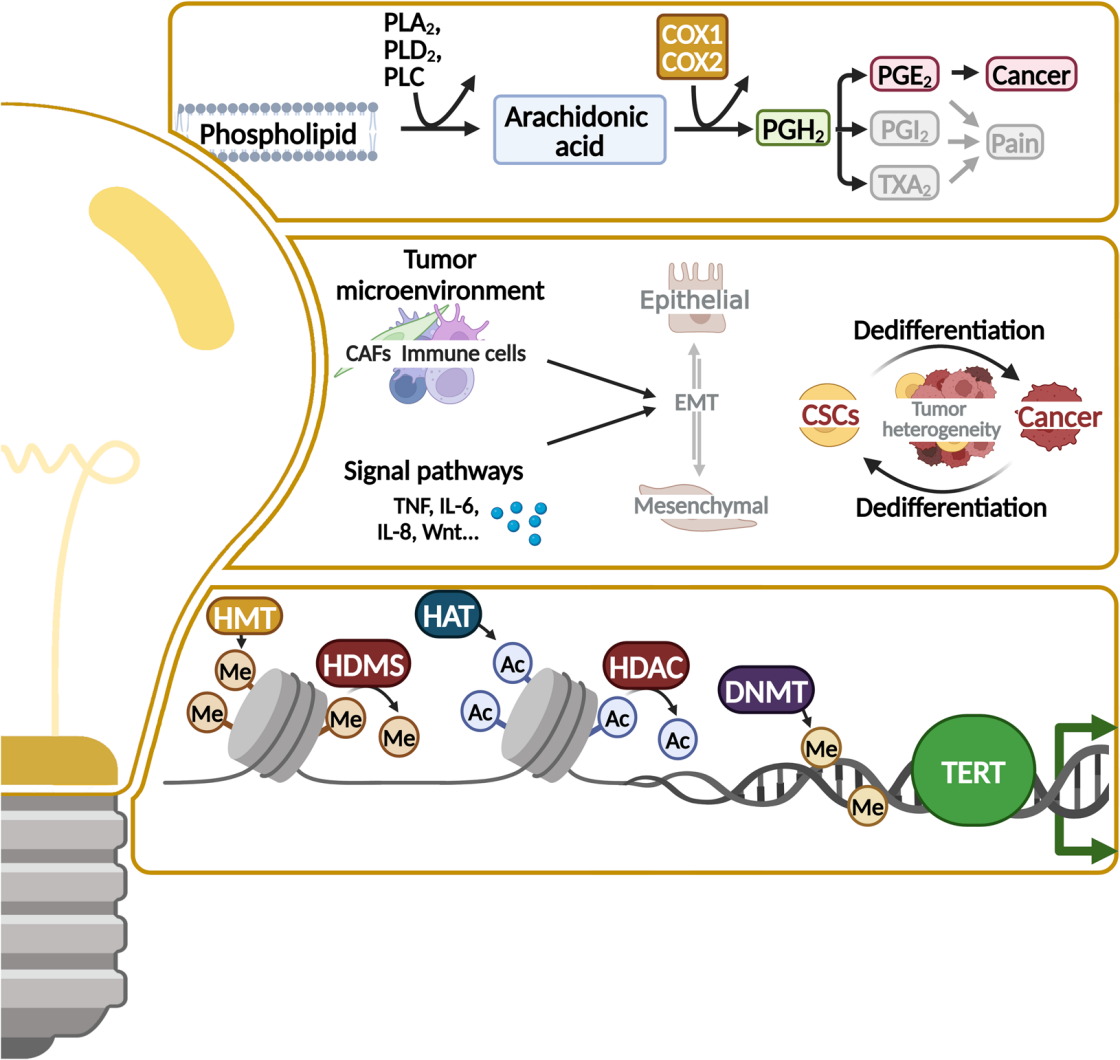
Further hallmarks to target. The hallmarks deregulating cellular metabolism, unlocking phenotypic plasticity and the enabling characteristics tumor-promoting inflammation offer broad possibilities of altered pathways to target by cancer treatment. Auspicious targets are displayed in the boxes. CSC, cancer stem cells; CAF, cancer-associated fibroblasts; TCA, tricarboxylic acid cycle; TERT, telomerase reverse transcriptase; HMT, histone methylases; HDMS, histone demethylases; HAT, histone acetyltransferases; HDAC, histone deacetylase; DNMT, DNA methyltransferase. This figure was created using Biorender.com

**Fig. 9:**
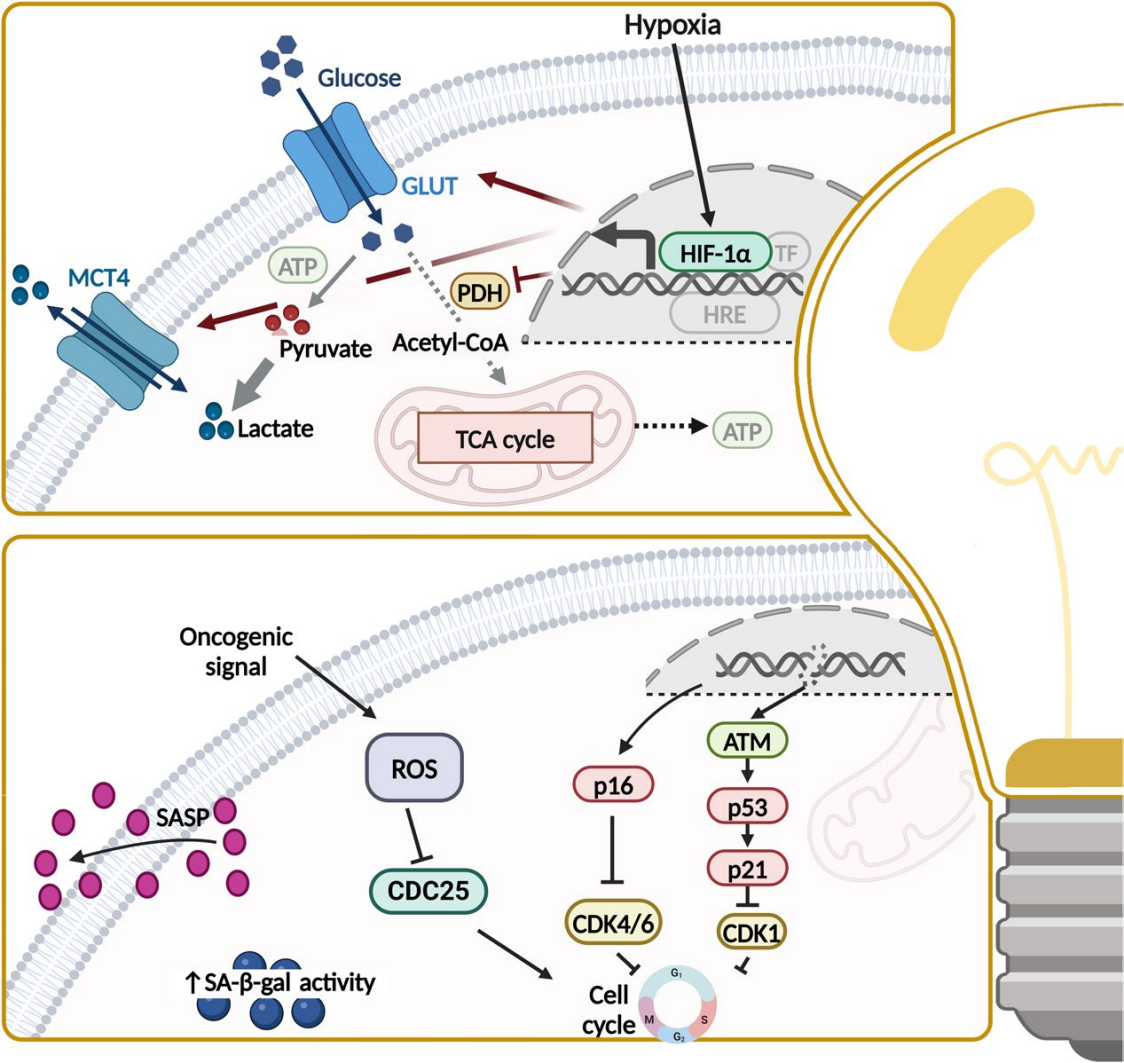
Further hallmarks to target. Other promising targets for treatment are the reprogramming of glucose metabolism in cancer cells and the cellular senescence of cancer by stimulating the senescence-associated secretory phenotype, which consists of proinflammatory cytokines, chemokines and matrix-reforming factors. MCT4, monocarboxylate transporter 4; ATP, adenosine triphosphate; GLUT, glucose transporter; TCA, tricarboxylic acid cycle; PDH, pyruvate dehydrogenase; HIF, hypoxia-inducible factor; ROS, reactive oxygen species; SASP, senescence-associated secretory phenotype; SA-β-gal, senescence-associated beta-galactosidase. This figure was created using Biorender.com

Since, to our knowledge, no phase III clinical trials have evaluated compounds targeting the hallmarks “enabling replicative immortality,” “deregulating cellular metabolism,” “senescent cells,” and “unlocking phenotypic plasticity,” nor targeting the enabling characteristics “tumor-promoting inflammation,” “nonmutational epigenetic reprogramming,” and “polymorphic microbiomes,” we will discuss current preclinical strategies and initial ongoing clinical trials that indicate possible future directions (Figs. 8 and 9).

Infiltration of TME by inflammatory cells promotes neoplastic progression by the supply of growth factors, survival factors, extracellular matrix-modifying enzymes, and angiogenic molecules (Fig. 8) [14]. Cyclooxygenases (COX), especially COX-2, are fundamental in induction of inflammatory state, which has been proven to come along with poor outcome in OC patients [20, 238]. Preclinical studies have demonstrated that high COX-2 activity increases cell migration and cisplatin resistance in OC cells, explaining promising activity (ORR 28.9%) of *celecoxib* (COX-2 inhibitor) and carboplatin combination in a phase II study [239]. Unfortunately, further studies investigating COX-inhibitors in combination with cytostatic drugs did not show differences in OS [240–242]. High COX-2 levels also correlate with a low response to immunotherapy and COX-2 inhibition reduced T_reg_ infiltration of the tumor [243]. Thus, *acetylsalicylic acid* (COX-1/2i) was combined with atezolizumab and bevacizumab in phase II study (EORTC 1508), but no efficacy benefit was observed [244]. Synergistic activity of TLR3 ligands, IFNα, and COX-2 inhibitors enhancing cytotoxic T-lymphocytes meanwhile suppressing T_regs_ was investigated in a phase I trial in recurrent PSROC [245]. Good safety and tolerability, as well as chemoattraction of cytotoxic T-lymphocytes by the triplet composed of cisplatin, *rintalomid* (TLR3 ligand), and celecoxib (+ in some cases IFNα as adjunct), led to a phase II trial investigating this triplet together with autologous tumor-loaded αDC1 vaccine. Unfortunately, PFS improvement did not meet predefined thresholds [246].

Whereas non-cancerous cells mainly process glucose to pyruvate and subsequently to carbon dioxide in mitochondria using the tricarboxylic acid cycle, cancer cells are mainly restricted to glycolysis. Since the “Warburg effect” is way less efficient in energy supply, upregulation of glucose transporters as GLUT1 and enhanced glutaminolysis can be seen in OC [14, 247–249]. Upregulation of GLUT1 is associated with poor prognosis in OC [250]. Key regulators of cancer cell metabolism are hypoxia-inducible factor 1α (HIF-1α) and AMP-activated protein kinase (AMPK). Oncogenes, tumor suppressors, and other signaling pathways as c-myc, RAS, p53, and AKT/PI3K/mTOR signaling further modulate energy metabolism (Fig. 9) [14, 20, 248]. *CRLX101* is a HIF-1α directed nanoparticle-drug conjugate transporting camptothecin (topo I-inhibitor) to cancer cells. Encouraging results in phase II trials as monotherapy and in combination with bevacizumab or paclitaxel in ROC merit future investigation [21, 251–253]. Besides, pan-AKT inhibitor *capivasertib* (AZD5363) reached clinical studies and has shown good tolerability and safety in phase I [254]. Recently capivasertib has been approved in metastatic hormone receptor positive breast cancer in combination with fulvestrant [255]. Combination of capivasertib and olaparib has shown great antitumor activity in phase I study including OC, likewise did preliminary results of a study investigating mTORC1/2 inhibitor *vistusertib* (AZD2014) in combination with olaparib evidence durable anti-tumor activity [256–258]. Other preclinical strategies inhibiting aerobic glycolysis include *BH3 mimetics*, *ivermectin*, *berberine*, and *ginsenoside* and are reviewed elsewhere [21, 249].

Telomer shortening arises with each cell division and is a natural barrier of replicative immortality (Fig. 8). Critical telomere attrition promotes extensive genomic instability, which leads to apoptosis via p53 and RB pathway or to replicative senescence. Cancer cell alterations such as loss of TP53 and restoration of telomerase activity enable survival of incipient malignancies [14, 259]. Ninety percent of cancers are characterized by overexpression of telomerase, which counteracts telomere attrition by its telomerase reverse transcriptase (TERT) [260, 259]. Cancers with TERT promoter mutations and high expression of TERT are associated with poor outcome, making them and the telomer shortening, an interesting drug target [259, 261]. Current approaches consider small-molecule telomerase inhibitors, oligonucleotide inhibitors, telomerase-directed gene therapy, immunotherapeutic approaches, and alternative splicing as treatment [261]. Further approaches include the attack of shelterin complex and targeting of alternative lengthening of telomeres [261]. However, only *imetelstat* (GRN163L), an inhibitor of telomerase activity, has been tested in advanced clinical studies for myelodysplastic syndrome and non-small cell lung cancer [262]. Other preclinical hopefuls include *BIBR 1532* (telomerase inhibitor) and *pyridostatin* (G-quadruplex stabilizer), which have shown promise in tumor spheroids [261, 263, 264].

“Senescent cells” were described as a new hallmark of cancer in 2022. Senescence is a non-proliferative but viable state of cells, concomitant with changes in cell morphology and activation of senescence-associated secretory phenotype (SASP) releasing chemokines and cytokines supporting proliferative signaling, angiogenesis, and metastasis (Fig. 9) [14, 15, 265]. Induction of senescence varies by DNA damage, imbalances in cell signaling, and cellular stress [15, 265]. SA-β gal, p16, and p21 represent several biomarkers of cellular senescence, but only a few markers have high specificity and sensitivity [266]. Some senescent cells can regain replicative abilities by cellular plasticity, considering senescence as a mechanism of therapy resistance [267]. Therefore, senescence has a key importance not only for tumor development but also for the response to cancer therapy and is correlated with poor prognosis [268, 269]. Thus, targeting senescence with senolytic or senomorphic drugs, as well as stem cell therapies, was able to extend lifespan and to minimize tissue damage in various animal models [266]. Combination with other anticancer drugs contributes to overcome resistance to apoptosis and reduce side effects [266]. For example, high expression of Bcl-x(L) induces senescence-mediated chemoresistance, which can be reduced by BCL-2 inhibitors as *navitoclax*, which was proven in phase II MONA VI-1 trial [116, 270]. According to epidemiological data, *metformin* is protective in OC and modulates the SASP, inhibits endothelial senescence, and enhances efficacy of CDK4 and CDK6 inhibitors [266, 271]. Furthermore, the inhibition of DYRK1A/B and DREAM complex, which are involved in cellular senescence in OC, is promising [271, 272]. Since hyperactivation of AKT/PI3K/mTOR signaling in OC is common, its inhibition is broadly investigated for OC treatment. Interestingly, it has been shown that AKT inhibition promotes senescence of cancer cells. Therefore, combination of AKT inhibitors and downstream blockage of autophagy and senescence could help to overcome therapy resistance [271]. Inversely, maintenance therapy with AKT inhibitors could keep tumor cells in senescent state, thereby preventing tumor recurrence [271]. However, in general, it remains controversial whether cellular senescence impedes cancer growth or supports tumor progress via SASP [15, 273]. In this context also, cancer stem cell (CSC)-related cell senescence displays an interesting approach to target [274].

Unlocking phenotypic plasticity covers another newly introduced hallmark of cancer, the ability to escape or evade terminal differentiation (Fig. 8). Cancer can acquire new molecular properties through dedifferentiation, blocked differentiation, or transdifferentiation, which facilitate metastasis and evasion of systemic therapy [15]. Influencing mechanisms are EMT, the formation of cancer stem cells, the activation or suppression of important signaling pathways, epigenetic changes, and changes in the tumor environment [275]. There is proof that EMT serves as a protective mechanism for cancer cells to survive. By inhibition of EMT, cisplatin resistance was successfully overcome in OC [275–277]. IL (interleukin)−8 contributes to tumor cell remodeling and is taking part in the regulation of tumor cell stemness, EMT, and resistance to therapy [275]. Treatment with *SB225002* (CXCR2 inhibitor) attenuates IL-8-induced resistance in OC cells [278]. As therapy-related resistance is still a major obstacle to a complete cure, it is crucial to understand the mechanisms involved in plasticity, to develop targeted therapies [275].

Epigenetic alterations, such as histone modifications, DNA methylation, and post-transcriptional modifications of RNA, influence gene expression and promote tumor development [15, 279, 280]. Several epigenetic alterations can be used as predictive markers in molecular cancer screening and to derive treatment recommendations [281]. Dynamic epigenetic changes in cancer are related to unlimited self-renewal and multi-lineage differentiation as well as tumor heterogeneity and display possible escape mechanisms to therapy which are druggable [15]. MicroRNAs (miRNA), small non-coding RNAs regulating gene expression organized in tumor suppressive or oncogenic clusters, are described in pathogenesis of OC and correlate with therapy response as well as patients’ outcome and serve as biomarkers [282, 283]. For example, Let-7 family of miRNA has a tumor suppressor function and is downregulated in many cancers. Let-7 g overexpression induces a significant reduction in OC cell growth [283]. Aberrant CpG island methylation in OC influences apoptosis, drug sensitivity, and cell cycle regulation. Prime example of aberrant methylation in OC is BRCA1 silencing by promoter hypermethylation [281]. Other epigenetic mechanisms, as shown by *ep-100* which targets gonadotropin-releasing hormone receptor and combined with olaparib increases histone H2A.X phosphorylation or the gain of platinum sensitivity due to *USP-1 inhibitors*, which stop deubiquitination of SNAIL, need to be further explored [281]. DNA methyltransferase (DNMT), histone deacetylase (HDAC), histone demethylase (HDT), and histone methyltransferase EZH2 are the main targets of so far marketed epidrugs [281]. Among DNMTis, *ginsenoside Rg3* have shown to promote apoptosis; *guadecitabine* (SG-110) have increased PARPi sensitivity regardless of BRCA status [281, 284]. Current limitations of DNMTis are mainly due to toxic side effects [281]. HDACi, such as *romidepsin*, *vorinostat*, *valproate*, and *PDX101*, induce acetylation in OC and thereby promote transcriptional activation and synergism with platinum-based therapies [281, 285, 286]. *Roxyl-ZHC-84* is a new HDACi, impeding JAK1-STAT3-BCL-2 provided resistance mechanism [287].

Evidence is growing that microbiota which are symbiotically associated with multiple barrier tissues impact cancer phenotype by either cancer-protective or tumor-promoting microbiome [15, 288, 289]. Mutagenesis due to bacterial toxins, epithelial proliferation caused by ligand mimetics and altered immune response and barrier function are ways how the microbiome can affect cancer. In addition, microbes can trigger DNA damage and apoptosis by releasing genotoxic metabolites or by formation of reactive oxygen species. In OC patients, cytokine levels of tumor necrosis factor α (TNFα) and IL-6, which are involved in regulation of tumor progression via JAK/STAT3 pathway and are regulated by gut microbiome, were increased [290, 291]. Also, a link between chlamydia infections and the risk of OC has already been established. Further endeavors employ microbiome alterations as biomarkers for OC [292]. Recently, Choi and Choi have described the role of the gut and cervicovaginal microbiota in OC concomitant with new therapeutic approaches, including, among others, *fecal microbiome transplantation* and *vaginal microbiome transplantation* to improve patients’ outcome [292–294].

## Targeted transport

**Fig. 10:**
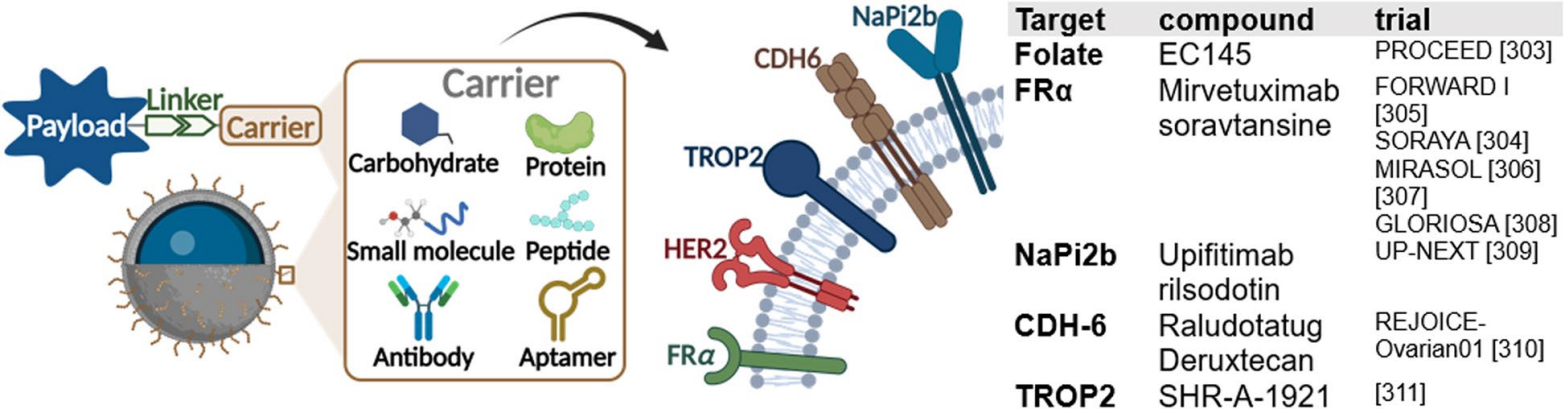
Targeted transport—overexpressed cell surface markers. Antibody drug conjugates (ADC) are composed by a carrier, a payload, and a linker. Common carrier molecules include carbohydrates, proteins, small molecules, peptides, and aptamers. FRα, HER2, TROP2, CDH6, and NaPi2b represent overexpressed surface molecules of OC, which are possible targets for ADCs. Recently completed and currently ongoing studies investigating ADC for OC are listed in the table. This figure was created using Biorender.com [302–310]

Targeted drug delivery systems that bind antibody, peptide, polymer, small molecules, and single-stranded oligonucleotides via linker enable selective delivery of high potent cytostatic drugs, thus increasing efficacy while decreasing systemic toxicity [295, 296]. A range of overexpressed surface markers is known for OC, including FRα, trophoblast antigen 2 (TROP2), cadherin 6 (CDH6), and type II sodium-phosphate cotransporter (NaPi2b) (Fig. 10). Preclinical research works at terrific rate on systematic identification and validation of further markers [295, 297]. After binding, the system usually passes the cell membrane by endocytosis, receptor-mediated uptake, or non-endocytic translocation pathways and thus reaches its target [298].

So far (03/24), 13 antibody drug conjugates (ADCs) have been approved worldwide [299]. *Mirvetuximab soravtansine* (MIRV), an FRα-directed antibody linked to maytansinoid DM4, is the first and only ADC approved by the FDA for OC [300]. FRα is overexpressed in about 90% of OC and expression increases with progress of disease [301]. A folate, conjugated with a vinca alkaloid, *vintafolide*, was already investigated in the phase III PROCEED trial in 2011 but did not meet the futility threshold [302]. In contrast, MIRV has proven high efficacy in clinical trials, such as SORAYA study (phase II/III) with an ORR of 32.4% [303].

MIRV is the first treatment demonstrating a benefit in PFS (HR 0.65) and OS (HR 0.67) in PRROC, which has been shown in MIRASOL study, confirming the good therapy response seen in SORAYA [305]. In November 2022, FDA granted accelerated approval for patients with FRα positive PRROC, who have previously received one to three systemic therapies [11]. A global study, GLORIOSA, is currently investigating the maintenance therapy in combination with bevacizumab in PSROC [307].

NaPi2b overexpression is reported in 95% of OC [295]. *Upifitamab rilsodotin* (UpRi) is a promising ADC targeting NaPi2b, linked to an anti-mitotic drug [311]. However, the phase III trial UP-NEXT was stopped in 2023 due to severe bleeding [308, 312]. *Raludotatug deruxtecan* is a potential first-in-class ADC, targeting CDH6. CDH6 is overexpressed in approximately 65% of OC [313]. Linked to the topoisomerase (topo) I-inhibitor deruxtecan, it has shown acceptable tolerability and early signals of efficacy in heavily pretreated women with OC as shown in interim-analysis of phase I study in 2023 [314]. Based on this, phase II/III REJOICE-Ovarian01 trial was initiated in February 2024 [309]. TROP2, a transmembrane glycoprotein, is overexpressed in 47–89% of OC and its overexpression is associated with poor prognosis [315]. *Sacituzumab* *govitecan *(Trodelvy), a TROP2-directed ADC, is already approved for triple negative breast cancer [316]. In *SHR-A-1921*, a topo I-inhibitor is connected to a TROP2 antibody with a cleavable linker [317]. Based on good safety and efficacy profile in a phase I trial, a phase II/III trial started in February 2024, evaluating the benefit in combination with carboplatin in ROC [310]. In addition, the TROP2 directed ADC *BNT325/DB-1305* is clinically promising and received FDA fast track designation for PRROC [318].

Further endeavors to optimize targeted drug delivery encompass bispecific ADCs [298]. One representative in preclinical research is the novel *SORT1xHER2* bispecific ADC, which is directed against HER2 and sortilin-1, which are co-overexpressed in OC [319]. Nevertheless, challenges regarding ADC, such as limited drug-to-antibody ratio and antibody-induced immunogenicity, remain [295].

Furthermore, there are drug-loaded nanoparticles that diffuse to the tumor tissue due to enhanced permeabilization and retention effect [295, 320, 321]. An example of this is liposomal doxorubicin (Doxil), a non-targeted nanoparticle approved by the FDA in 1995 [322]. Nanoparticle drug systems in clinical trials for OC include *EP0057*, camptothecin bound to a cyclodextrin-based polymer scaffold, and *ELU001*, exatecan combined with folic acid analogs [251, 323].

## Further approaches

Intra- and intertumoral heterogeneity (morphological, prognostic, etiopathogenetic, and molecular heterogeneity) as well as growing knowledge about the impact of TME on cancer emphasize the importance of prognostic screening methods for treatment response, e.g., patient-derived ex vivo tumor organoid cultures, patient-derived xenografts, or “tumor/organ on a chip” models [324, 325]. Future dream would be to predict therapy response solely based on tumor sequencing. Due to this heterogeneity, biomarkers, patient stratification or rather precision oncology and adequate monitoring are crucial to select the appropriate therapy for each patient and thus contribute to the success of the drug, as shown in the work by Skorda et al. [17]. Therefore, not only tests are needed to identify subgroups but also markers with higher specificity and sensitivity. Furthermore, the individualized therapy approach is progressively represented in molecular tumor boards in the clinics.

Besides the development of new drugs, the investigation of *tumor-treating field* (TTF), an upcoming new cancer treatment modality, using alternating electric fields of intermediate frequency that are intended to disrupt tumor cell growth, is interesting [326]. INNOVATE-3, a phase III study, recently investigated TTF in combination with paclitaxel for PSROC. Although the primary endpoint of OS was not met, survival benefits among exploratory subgroups could be seen and merit further subgroup analyzes [327]. Taking into account major prognostic importance of complete debulking of OC for patient outcome, upcoming imaging agents as *Gleolan* (5-ALA) are another important tool to improve surgery [328]. Recently, a phase III study (OVA-302) was designed to investigate whether Gleolan can improve debulking surgery of OC [329, 330].

An appropriate study design is essential in order to be able to identify effects and side effects in clinical studies. This includes the choice of suitable biomarkers, the appropriate selection of in-process and follow-up controls, and the perfectly responsive subgroup, due to the large heterogeneity in OC. An incorrect selection of subgroups can lead to a reduction in the effect or even the absence of an effect. Another challenge in clinical trials is long-term follow-up, included in both phase III and phase IV trials. With the increase in patient cohort size and observation time, rare or slowly developing adverse effects are more likely to be detected. Pharmacovigilance is the monitoring of safety and/or efficacy over a longer-term period. Combination therapy is often used to reduce the risk of a poor safety profile. However, the challenge continues even after the clinical trials. In clinical trials, optimally suited patients are initially included in the study. Subgroups are optimally selected and intensively monitored. In the clinic, the drug is then used in a larger cohort of patients with poorer general health, different ages, and increased heterogeneity. For this reason, biomarkers, prognostic tests for treatment response (precision oncology), and appropriate monitoring are essential, even after approval, in order to be able to select the appropriate therapy for each patient and thus contribute to the success of the drug.

## Conclusions

OC remains the most lethal gynecological cancer. Challenges faced in OC regarding drug therapy remain to be drug resistance mechanisms and CSCs leading to relapse situations. Many new strategies to improve patient’s outcome appear upon the horizon. Using tools as targeted therapy, immunotherapy, gene therapy, and drug-conjugates, a variety of new techniques and compounds has been developed within the last years to target the hallmarks of cancer.

For example, kinase inhibitors have been broadly investigated in OC treatment. To face challenges such as intratumoral heterogeneity and alterations of multiple pathways, mainly combinational treatments are currently under clinical evaluation. Especially, regarding immunotherapy, huge improvement was made within the last years. Numerous new compounds have evolved and have been investigated, finally showing OS benefits in treatment with ICI and PARPi in the DUO-O trial.

Newly emerged hallmarks of cancer and enabling characteristics as phenotypic plasticity, epigenetic reprogramming, and the microbiome display interesting targets to treat. Here, development and investigation of new compounds is still at the very beginning and merits future research. The information gaps in clinical studies that exist due to general absence of a mandatory international uniformly study register and due to the lack of an obligation to publish results should also be closed in the future.

Following the breakthrough of bevacizumab and PARPi within the recent years, the ADC MIRV appears to be the next drug with great potential in the pipeline. However, we are eagerly awaiting the pending study results and are curious to see which strategies to improve OC therapy will prevail.

## Data Availability

Data for this review were identified by searching ClinicalTrials.gov (1) using keywords “ovarian cancer” “female” accessed: 03/19/2024 and PubMed and references from relevant articles using the search terms like “ovarian cancer,” “hallmarks of cancer,” “treatment,” “targeted therapy,” “chemotherapy,” “PARP inhibition,” “immunotherapy,” and/or “antibody drug conjugates.” No datasets were generated or analysed during the current study.

## Abbreviations

ATP: Adenosine triphosphate
ACT: Adoptive cell therapy
alt-EJ: Alternative end joining
AMPK: AMP-activated protein kinase
ALK: Anaplastic lymphoma kinase
AR: Androgen receptor
Ang: Angiopoietin
ADC: Antibody drug conjugates
BRCA: Breast cancer gene
CDH6: Cadherin 6
CAF: Cancer-associated fibroblasts
CSC: Cancer stem cell
CAR-T: Chimeric antigen receptor T cells
CHMP: Committee for Medicinal Products
CDKs: Cyclin-dependent kinases
COX: Cyclooxygenases
DLL4: Delta-like ligand 4
DCV: Dendritic cell vaccines
DC: Dendritic cells
DNMT: DNA methyltransferase
DSB: Double-strand breaks
T_Eff_: Effector T cells
EGFR: Epidermal growth factor receptors
EMT: Epithelial-mesenchymal transition
EMT-TF: Epithelial-mesenchymal transition transcription factors
ER: Estrogen receptor
FADD: Fas-associating protein with death domain
FGF: Fibroblast growth factor
FAK: Focal adhesion kinase
FRα: Folate receptor alpha
gBRCAm: Germline BRCAm
GLUT: Glucose transporter
HH: Hedgehog signaling pathway
HGSC: High-grade serous cancer
HAT: Histone acetyltransferases
HDAC: Histone deacetylase
HDT: Histone demethylase
HDMS: Histone demethylases
HMT: Histone methylases
HR: Homologous recombination
HRD: Homologous recombination deficient
HRP: Homologous recombination proficient
HIF: Hypoxia-inducible factor
IHC: Immunohistochemistry
ICVs: Infected cell vaccines
IAP: Inhibitors of apoptosis
IL: Interleukin
LGSC: Low-grade serous cancer
MHC: Major histocompatibility complex
miRNA: MicroRNAs
MIRV: Mirvetuximab soravtansine
MCT4: Monocarboxylate transporter 4
NK: Natural killer
NHEJ: Non-homologous end joining
OV: Oncolytic viruses
OC: Ovarian cancer
ORR: Overall response rate
OS: Overall survival
PDOs: Patient-derived organoids
PLD: Pegylated liposomal doxorubicin
PDGFβ: Platelet-derived growth factor β
PSROC: Platinum-sensitive recurrent OC
PRROC: Platinum-resistant or -refractory recurrent OC
PLK1: Polo-like kinase 1
PARPi: Poly ADP-ribose polymerase inhibitors
PD-L1: Programmed cell death ligand-1
PFS: Progression-free survival
PDH: Pyruvate dehydrogenase
ROS: Reactive oxygen species
ROC: Recurrent OC
T_reg_: Regulatory T cells
RB: Retinoblastoma-associated
SA-β-gal: Senescence-associated beta-galactosidase
SASP: Senescence-associated secretory phenotype
SSB: Single-strand breaks
TERT: Telomerase reverse transcriptase
TRADD: TNFR1-associated death domain protein
topo: Topoisomerase
TCA: Tricarboxylic acid cycle
TROP2: Trophoblast-antigen-2
TAAs: Tumor-associated antigens
TGF β: Tumor growth factor β
TIL: Tumor-infiltrating lymphocytes
TME: Tumor microenvironment
TNF: Tumor necrosis factor
TTF: Tumor-treating field
NaPi2b: Type II sodium-phosphate cotransporter
USP-1: Ubiquitin-specific protease 1
UpRi: Upifitamab rilsodotin
VEGFA: Vascular endothelial growth factor A
